# Chemical probes for competitive profiling of the quorum sensing signal synthase PqsD of *Pseudomonas aeruginosa*

**DOI:** 10.3762/bjoc.12.277

**Published:** 2016-12-20

**Authors:** Michaela Prothiwa, Dávid Szamosvári, Sandra Glasmacher, Thomas Böttcher

**Affiliations:** 1 Department of Chemistry, Konstanz Research School Chemical Biology, University of Konstanz, 78457 Konstanz, Germany

**Keywords:** activity-based probes, PqsD, protein labelling, *Pseudomonas aeruginosa*, quinolones

## Abstract

The human pathogen *Pseudomonas aeruginosa* uses the *pqs* quorum sensing system to coordinate the production of its broad spectrum of virulence factors to facilitate colonization and infection of its host. Hereby, the enzyme PqsD is a virulence related quorum sensing signal synthase that catalyzes the central step in the biosynthesis of the *Pseudomonas* quinolone signals HHQ and PQS. We developed a library of cysteine reactive chemical probes with an alkyne handle for fluorescence tagging and report the selective and highly sensitive in vitro labelling of the active site cysteine of this important enzyme. Interestingly, only one type of probe, with a reactive α-chloroacetamide was capable of covalently reacting with the active site. We demonstrated the potential of our probes in a competitive labelling platform where we screened a library of synthetic HHQ and PQS analogues with heteroatom replacements and found several inhibitors of probe binding that may represent promising scaffolds for the development of customized PqsD inhibitors as well as a chemical toolbox to investigate the activity and active site specificity of the enzyme.

## Introduction

**Figure 1:**
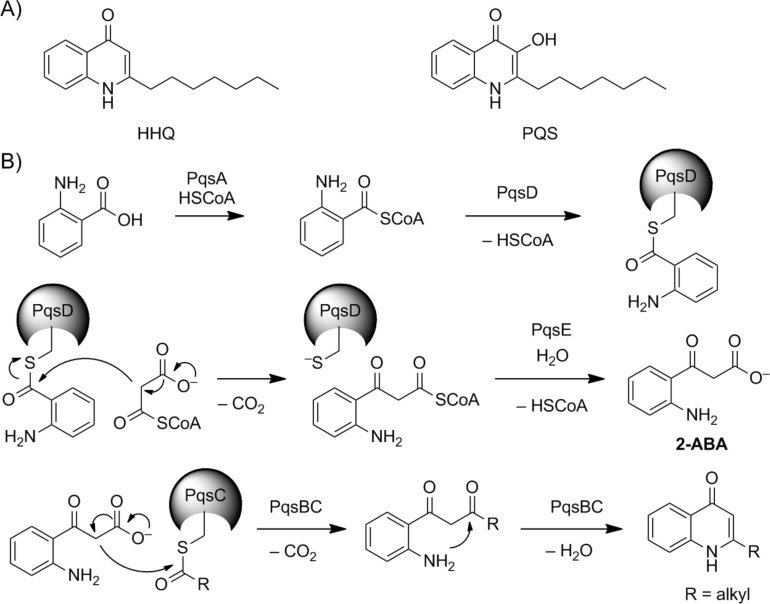
Quinolone signals of *Pseudomonas aeruginosa*. A) Structures of HHQ and PQS. B) Proposed mechanism for the synthesis of 2-alkyl-4-quinolones [12–14].

The emergence of multi-drug resistant bacterial strains urges the rapid discovery of new antibiotics and the development of novel antiinfective strategies [1]. One of the leading causes for nosocomial infections is the opportunistic human pathogen *Pseudomonas aeruginosa*, which, by chronic infections, also poses a major threat for cystic fibrosis patients [2–3]. *P*. *aeruginosa* deploys numerous virulence factors such as toxins, extracellular enzymes, and small molecule factors that are responsible for the bacterium’s ability to invade the host and cause a broad spectrum of different diseases [4–5]. The production of these virulence factors is coordinated on population level by several layers of hierarchically interconnected quorum sensing systems [6]. Quorum sensing signals are released from the cells and accumulate in a growing bacterial population to a certain threshold by which they start inducing the production of virulence factors. This simple signaling strategy thus regulates bacterial behaviour in dependence of population density. One of these quorum sensing systems, the *pqs* system, uses 2-alkyl-4-quinolones (AQs) as signals of which the Pseudomonas quinolone signal (PQS) and its biosynthetic precursor 2-heptyl-4-quinolone (HHQ) are the two best studied AQs (Figure 1A) [7]. A variety of virulence factors are under control of the *pqs* quorum sensing system, including the production of elastase, pyocyanin, PA-IL lectin, and rhamnolipids, as well as populations dynamic behaviours such as biofilm formation. However, the exact roles of the different AQs are still not completely understood [6,8].

Besides HHQ and PQS, in total more than 50 structurally related AQs have been detected in *P*. *aeruginosa* [9]. Key to this large diversity of natural AQs are their common biosynthesis steps by enzymes encoded in the *pqsABCDE* operon [10]. The biosynthesis of AQs has been matter of a long-standing debate that could only recently be resolved. Although HHQ could be produced in vitro by a PqsD catalyzed “head-to-head” decarboxylative Claisen condensation of activated anthranilic acid with β-keto fatty acid derivatives [10–11], isotope labelled feeding experiments indicated an entirely different mechanism for its biosynthesis [12]. This mechanism has been elucidated step by step in recent efforts by the work of various research groups. Hereby, PqsA activates anthranilic acid to anthraniloyl-CoA which is transferred to PqsD which catalyzes the condensation with malonyl-CoA to form 2-aminobenzoylacetyl-CoA. The thioesterase PqsE hydrolyses the thioester to produce 2-aminobenzoylacetate (**2-ABA**) [13]. The PqsBC complex finally generates HHQ or other AQs in a decarboxylative condensation reaction of **2-ABA** with fatty acids loaded on PqsC (Figure 1B) [14].

For the condensation step of an anthraniloyl residue with malonyl-CoA by PqsD, a cysteine residue (Cys112) is involved in the formation of a covalent thioester intermediate. We were speculating that activity-based electrophilic probes may be applicable to target this enzyme in vitro which could allow to study its active site reactivity in greater detail and apply a competitive labelling platform to discover potential PqsD inhibitors.

## Results and Discussion

### Electrophilic activity-based probes

The primary structure of PqsD comprises in total six cysteines. However, only one of them, Cys112, is engaging in the catalytic process forming a covalent reaction intermediate. We thus aimed at exploring the possibility to selectively label the active site cysteine residue using chemical probes.

Activity-based protein profiling (ABPP) has become a powerful tool to study protein function and elucidate targets of protein-reactive natural products in complex proteomes [15–18]. Various types of probes with an electrophilic core have been applied as tools for in vitro and in situ experiments of activity-based protein profiling [19–21]. ABPP uses probes with a reactive chemical group selectively targeting the active site of an enzyme and a reporter group that allows in-gel imaging and/or affinity enrichment of target enzymes [22].

**Figure 2:**
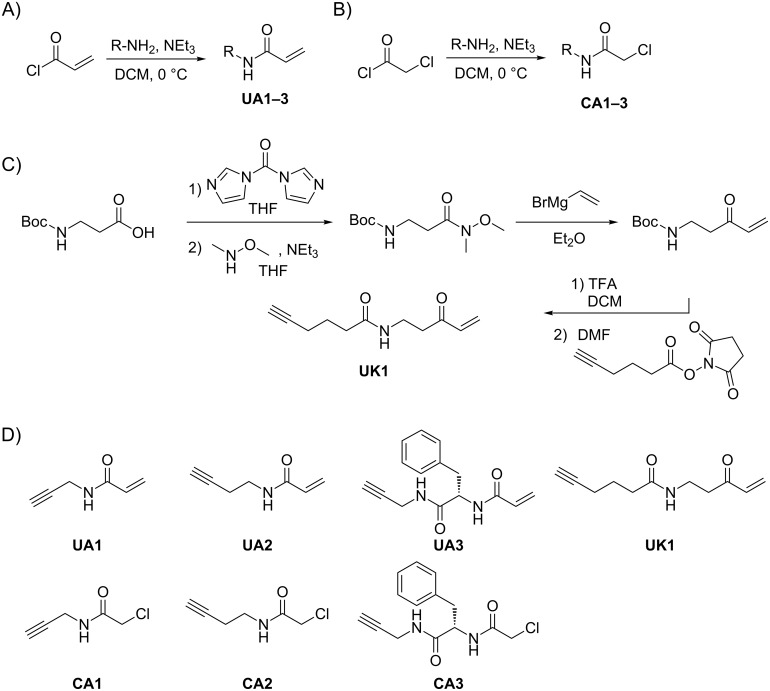
Synthesis of electrophilic ABPP probes. A) Synthesis of α,β-unsaturated amide probes **UA1–3**. B) Synthesis of α-chloroacetamide probes **CA1–3**, and C) synthesis of α,β-unsaturated ketone **UK1**. D) Structures of the ABPP probe library.

We thus synthesized a small library of chemical probes with electrophilic α-chloroacetamide, α,β-unsaturated amide, and α,β-unsaturated ketone moieties as protein reactive groups, which have been reported to exhibit selectivity for active site cysteines [19] (Supporting Information File 1, Figure S1). Each probe was equipped with a terminal alkyne handle for in-gel analysis by fluorescence tagging via click chemistry with a corresponding rhodamine azide. Variations of linker length and side group decorations between the reactive group and the alkyne handle were introduced to investigate potential differences in selectivity. Different alkyne amines were used to generate α,β-unsaturated amide probes **UA1–3** by reaction with acrylic acid chloride (Figure 2A) and α-chloroacetamide probes **CA1–3** by reaction with chloroacetyl chloride (Figure 2B). The α,β-unsaturated ketone probe **UK1** was synthesized via the Weinreb–Nahm amide in a Grignard reaction with vinylmagnesium bromide (Figure 2C). An overview of the small ABPP probe library is given in Figure 2D.

### Active site specific labelling of PqsD

**Figure 3:**
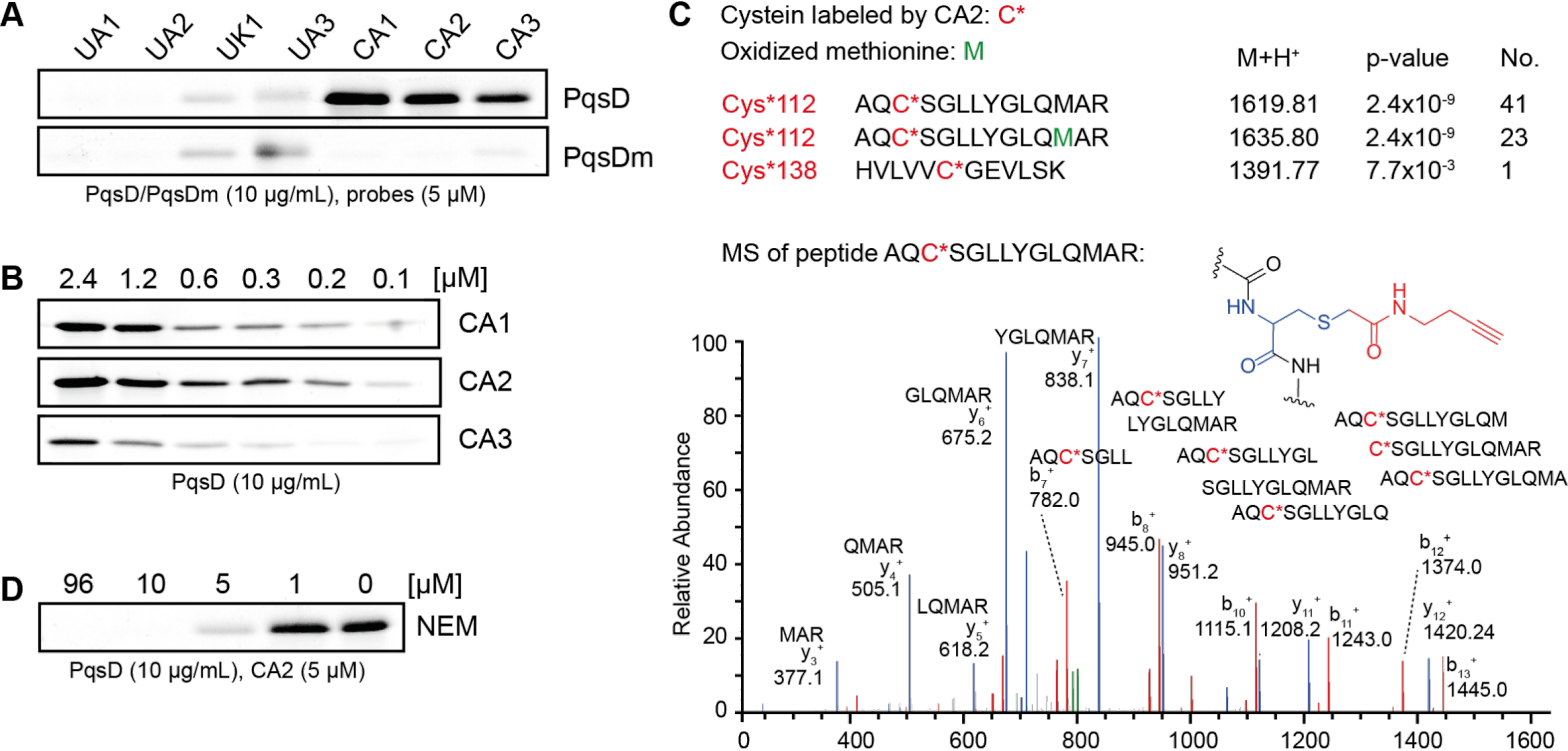
In vitro labeling of PqsD by chemical probes. A) ABPP probe library with wild-type PqsD and PqsD C112A mutant (PqsDm). B) Concentration dependence of labeling by the three active site directed probes. C) Mass spectrometric discovery of tryptic peptide fragments with probe **CA2** attached to the active site Cys112 (No.: number of detected peptides). D) Competitive experiment with *N*-ethylmaleimide and probe **CA2**.

Next, we were interested to investigate if any of the ABPP probes was capable of labelling PqsD. Therefore, we cloned the *pqsD* gene of *Pseudomonas aeruginosa* PAO1 into an expression vector encoding an N-terminal strep-tag. The protein was heterologously expressed in *Escherichia coli* BL21 followed by affinity purification by an ÄKTA chromatography system equipped with a StrepTrap HP column. The individual probes were incubated with purified PqsD for 30 min and a rhodamine fluorescent reporter tag was appended by click chemistry. The remaining non-covalently bound probe and excess reporter tag were removed by SDS-polyacrylamide gel electrophoreses (SDS-PAGE), and labelling of the protein was visualized by fluorescence imaging. Consistency of protein levels was checked by coomassie staining (Supporting Information File 1, Figures S2 and S3). While the α,β-unsaturated amide probes **UA1–3** and the α,β-unsaturated ketone **UK1** only resulted in very weak or no labeling, all three α-chloroacetamide probes **CA1–3** gave a strong fluorescent signal in the gel (Figure 3A).

In order to investigate the selectivity of the probes, we constructed a PqsD C112A mutant, where the active site cysteine was replaced by alanine. The purified mutant PqsD C112A exhibited only low background labeling for some probes but not comparable to labelling of the wild type protein by **CA1–3**, indicating that the probes were selectively targeting the active site (Figure 3A). Concentration series with a dose-down of the three CA probes showed that labelling of PqsD was concentration dependent and the two most potent probes **CA1** and **CA2** resulted in significant labelling at concentrations as low as 200 nM. Mass spectrometric analysis of a tryptic digest of **CA2** labelled wild type PqsD resulted in an additional mass corresponding to a probe modified cysteine residue Cys112 confirming that the CA probes indeed covalently labelled the active site cysteine. Only one peptide was detected with another cysteine residue (Cys138) modified by the probe compared to 64 detected peptides for **CA2** labelled Cys112 underlining the selectivity of our probes (Figure 3C). These results indicate that probes **CA1–3** are specific and covalently bind to Cys112 of PqsD and are thus, to the best of our knowledge, the first account of activity-based probes targeting and selectively labelling the active site of PqsD.

Interestingly, variations in the probe structure had little impact on labeling intensity and specificity. Although each α-chloroacetamide probe had one closely related α,β-unsaturated acetamide counterpart, only the reactive group but not the structure of the probe or its side groups determined active-site labeling. These findings are surprising, as all three reactive groups are known to bind to cysteines which indicate a fine-tuned nucleophilic reactivity of the active site cysteine Cys112. The fine-tuned nucleophilicity towards our probes is supported by calculations of a mechanistic model where Cys112 is activated by deprotonation by His257 [23]. Our results may also partially explain the potent inhibition of a PqsD inhibitor described in the literature which was discovered in silico and had been equipped with an α-chloroacetyl group [24].

Inhibition of PqsD has been proposed as promising antivirulence strategy leading to disruption of AQ signaling and thereby to global down-regulation of virulence factor production [11]. Consequently, PqsD has become a highly attractive target and a great amount of work pioneered by the Hartmann group has resulted in inhibitor discovery using a combination of in vitro assay, in silico modelling and chemical lead optimization. Examples of successful inhibitors are represented by the scaffolds of various 2-benzamidobenzoic acids [11,25–26], 2-nitrophenyl derivatives [27–29], ureidothiophene-2-carboxylic acids [24,30], and catechol-based compounds [31].

Many promising in vitro inhibitors based on these leads have been described and importantly, some of them also displayed in situ activity by reducing signal production and biofilm formation in live cultures of *P*. *aeruginosa* [28,31]. Recently, a synergistic dual PqsD and PqsR inhibitor was developed which also led to a marked decrease in the production of the virulence factors pyocyanine and pyoverdine [32].

So far only laborious enzyme-based assays, docking studies or modelling resulted in new scaffolds. We were thus interested, if our probes could be applied as a simple tool to discover novel scaffolds or chemical PqsD-binding motifs.

### Competitive screening approach

In order to test if our probes could be used as a competitive labelling platform, we used the well-known unspecific cysteine reactive agent *N*-ethylmaleimide (NEM) and dosed NEM in increasing concentrations to wild-type PqsD before applying the probe **CA2** followed by click chemistry with the fluorophore. A decreasing labelling intensity at increasing NEM concentrations indicates that NEM also blocks the active site cysteine Cys112 at concentrations above 5 µM and thereby prevents covalent attachment of the probe to PqsD (Figure 3D).

We were thus interested to investigate if we could demonstrate the value of our activity-based probes in a competitive screening approach by identifying potentially new scaffolds for PqsD inhibitors. We have recently reported the discovery of inhibitors of the virulence factor elastase of *P*. *aeruginosa* by a library of synthetic HHQ and PQS derivatives with systematic heteroatom replacements [33]. Because the interactions of PqsD with AQs is still not entirely understood, we reasoned that HHQ or PQS analogues may be promising scaffolds for inhibitor development and we thus aimed to screen this library competitively against the active site specific probe **CA2**. Therefore, we further refined the library by the synthesis of two additional HHQ analogues and implemented improved synthetic strategies.

**Scheme 1:**
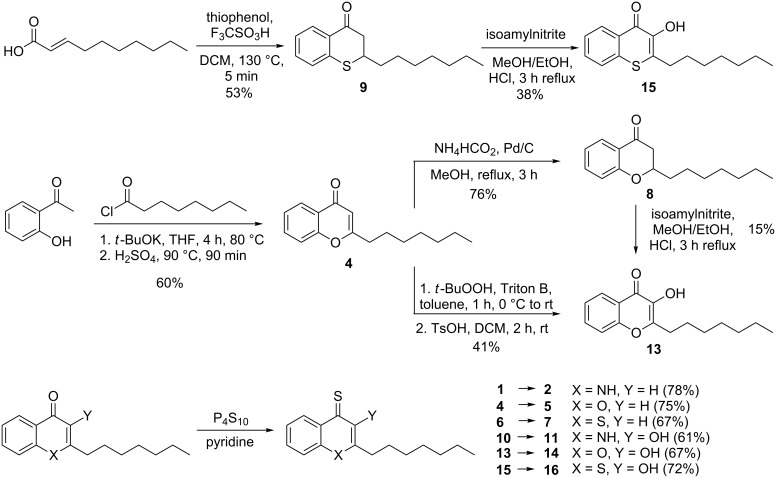
Synthesis of various HHQ and PQS analogues.

In detail, we synthesized 2-heptylquinolin-4(1*H*)-one (HHQ, **1**) and 2-heptyl-3-hydroxyquinolin-4-one (PQS, **10**) according to the described procedures by McGlacken et al. [34] and Hradil et al. [35], respectively. We previously described the synthesis of HHQ and PQS derivatives with nitrogen in position 1 exchanged by oxygen and sulfur [33]. In our efforts to optimize the synthesis of these heteroatom derivatives we used a one-pot reaction for the synthesis of 2-heptyl-chromen-4-one (1-O-HHQ, **4**) which includes esterification, Baker–Venkataraman rearrangement and subsequent acid-catalyzed ring closure to affort the 1-O-HHQ in 60% yield [36] (Scheme 1).

**Figure 4:**
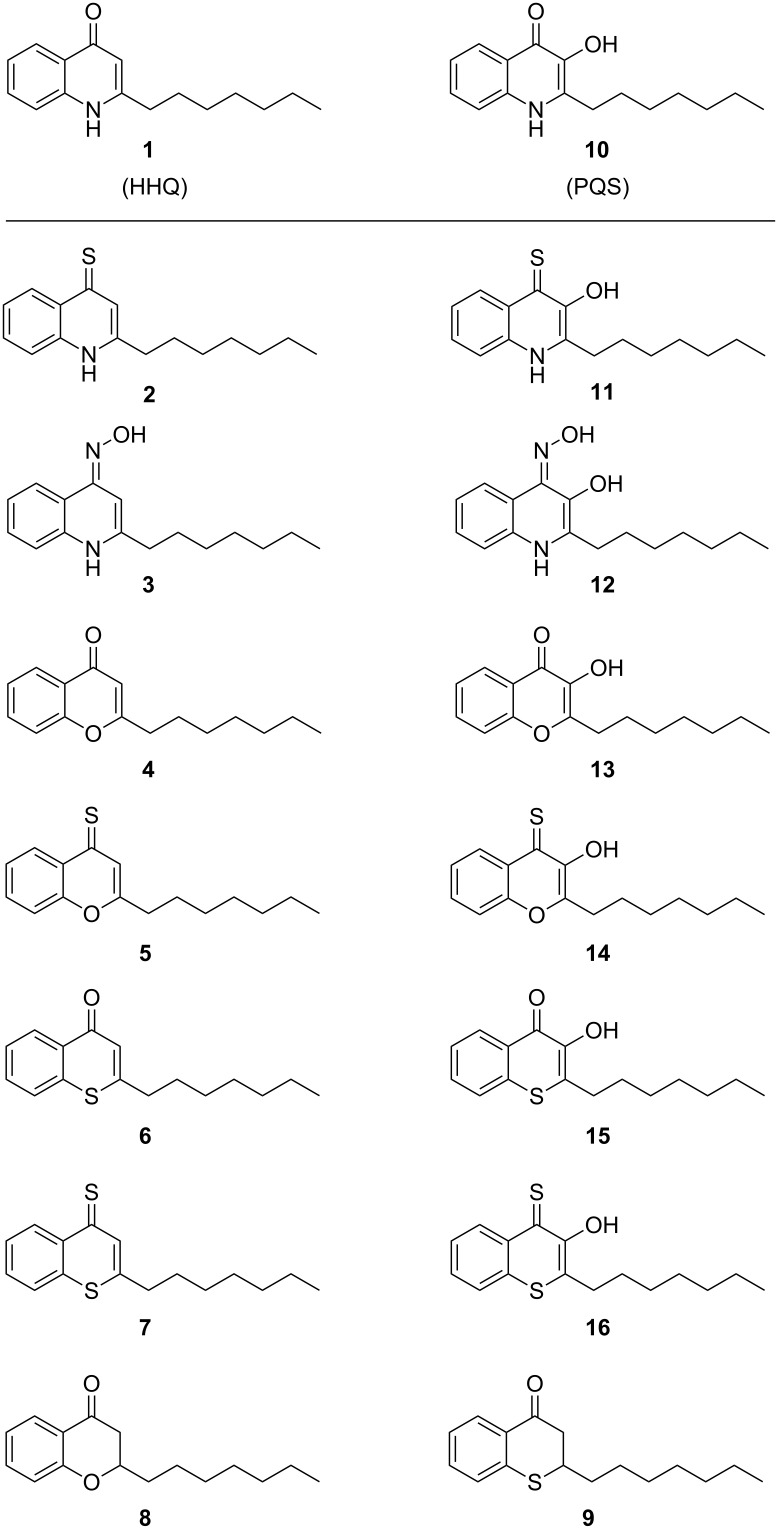
Library of HHQ and PQS analogues.

The 2-heptyl-3-hydroxychromen-4-one (1-O-PQS, **13**) was previously synthesized from the chroman-4-one **8** which was produced by base-catalyzed Knoevenagel reaction from 2-hydroxyacetophenone with octanal. Although the starting material was readily available, the reaction gave the product only in low yield (20–30%) and separation of the starting material from the product could be difficult especially for multigram scale approaches. Since 1-O-HHQ (**4**) was now easily available, we used **4** as starting point for the 1-O-PQS (**13**) synthesis by two different approaches. First, we tried to synthesize chroman-4-one **8** by hydrogenation of **4**. We found that ammonium formate (NH_4_HCO_2_) as mild hydrogen source with Pd/C gave chroman-4-one **8** in a clean reaction with a good yield of 76% whereas the direct use of H_2_ with Pd/C gave mainly the fully reduced 2-heptylchromane. Compound **8** can be applied for the synthesis of 1-O-PQS (**13**) as described in [33]. A new, direct way in which 1-O-HHQ (**4**) can be used for the synthesis of 1-O-PQS (**13**) was explored by epoxidation with subsequent ring opening in 41%. Thus, 1-O-PQS (**13**) could be produced in just two steps with an overall yield of 25% (Scheme 1). The synthesis of 1-S-PQS (**15**) was previously accomplished by a two-step synthesis of thiochroman-4-one **9** and following oxidation to give 1-S-PQS (**15**) in 12% overall yield [33]. In our attempt to synthesize **9** more efficiently, we used a method described by Olah et al. [37] starting from commercially available (*E*)-dec-2-enoic acid and thiophenol which gave **9** in 53% yield without the use of microwave assistance (Scheme 1). Thionation of the 4-position of **1**, **4**, **6**, **10**, **13** and **15** using P_4_S_10_ in pyridine under reflux conditions gave the 4-thiones **2**, **5**, **7**, **11**, **14** and **16** in yields between 60–80%, respectively (Scheme 1). The HHQ-oxime (**3**) was synthesized from HHQ (**1**) by conversion in the benzyl-protected chinolinol form (**3a**) and oximation with hydroxylamine hydrochloride similar to the described method used for the synthesis of the PQS-oxime **12** [33]. The entire compound library of HHQ and PQS analogues is presented in Figure 4. Further details on the syntheses are given in the Supporting Information File 1.

**Figure 5:**
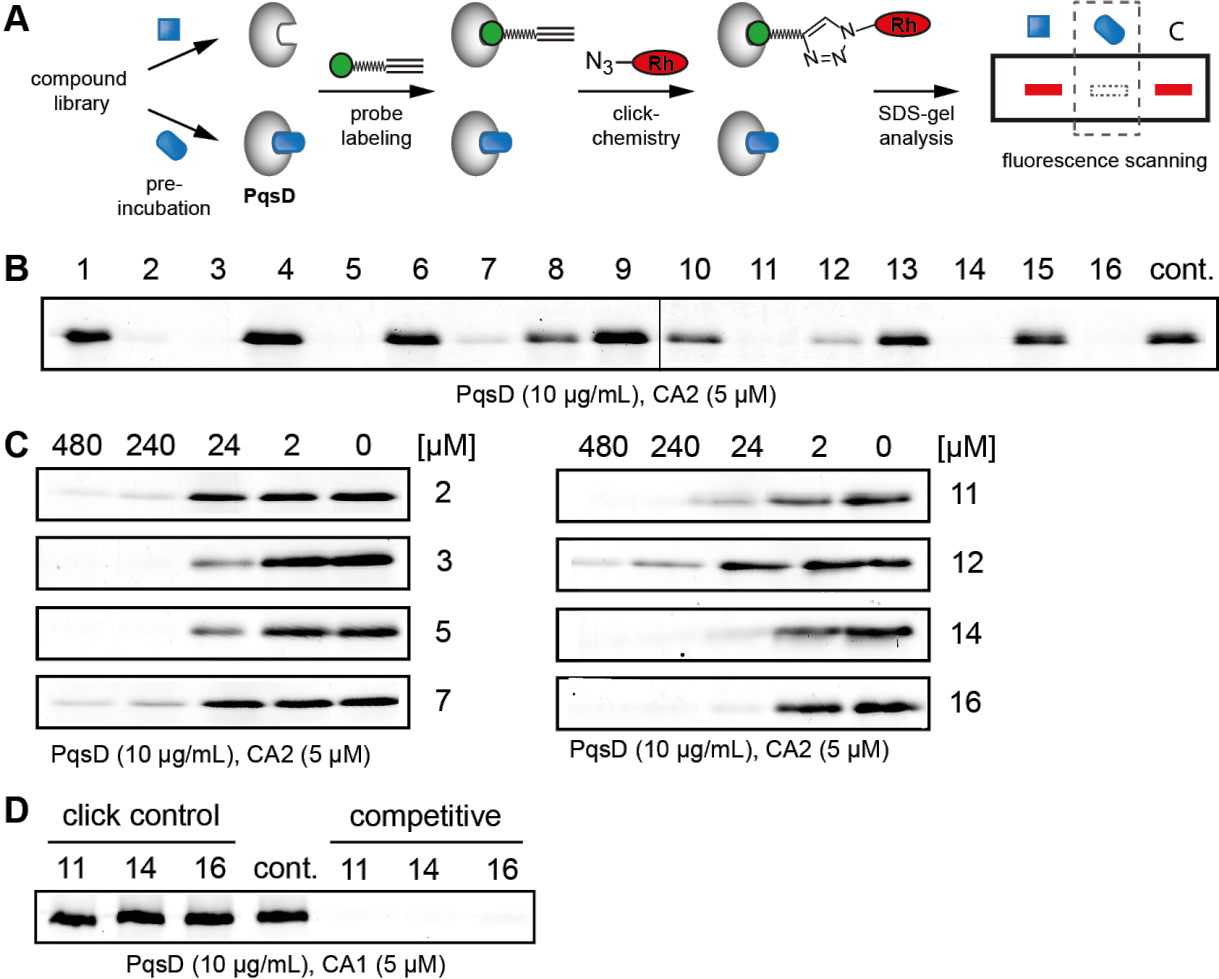
Competitive profiling platform. A) Schematic representation of the competitive labelling strategy with an alkyne probe (green) and potential inhibitors (blue). Rh = rhodamine. B) Initial screening of our small library of HHQ (**1**–**9**) and PQS (**10**–**16**) analogues against the active site specific chemical probe **CA2**. C) Concentration dependent competition experiment where the probe concentration is held constant and PqsD is pre-treated with varying inhibitor concentrations. D) Click chemistry control where the compounds were added shortly before Cu^II^-salt addition in the click protocol. Cont.: DMSO control.

All compounds were screened at an initial concentration of 240 µM in a competitive experiment against probe **CA2**. With the compounds added as DMSO stocks, solubility of the compounds was not an issue at these concentrations. PqsD was hereby pre-incubated with the compounds for 30 min, followed by the addition of the probe. A compound interacting tightly with the active site would hinder the probe from binding to the active site. Thus, a reduced labelling intensity in a competitive screening experiment indicates a potential hit compound (Figure 5A).

Strikingly, half of the derivatives were significantly active in the competitive screening and abolished probe labelling at 240 µM, while the other compounds had no such effect at this initial concentration (Figure 5B). Active compounds were found in pairs of HHQ and their corresponding PQS derivatives and either comprised a 4-thionated HHQ (**2**, **5**, **7**) or PQS (**11**, **14**, **16**) scaffold or an oxime group in position 4 (**3** and **12**).

The eight active compounds were tested in a concentration-dependent experiment in order to assess their potency in inhibiting probe binding. Interestingly, the HHQ derivative **3** with an oxime group was significantly more active than its PQS counterpart **12**, with the lowest activity. In contrast, the 4-thionated PQS derivatives **11**, **14** and **16** were always more active than their corresponding HHQ analogues (**2**, **5**, and **7**), and efficiently blocked probe labelling already around 24 µM. These results indicate that the 3-OH group was important for the activity of the 4-thionated compounds. In order to exclude any adverse effects of the compounds on the click chemistry, we performed control experiments where the most active derivatives were added directly before the last step of the click protocol. Intense labelling in this control group indicated that click chemistry was not affected by the compounds (Figure 5D). To assess the stability of the probe, we incubated **CA2** with two of the most active compounds, **11** and **14**. NMR and MS data indicate that the probe was not chemically modified even after 18 and 24 h so that the potential inactivation of the probe by the compound scaffold during protein labelling could be ruled out (Supporting Information File 1, Figures S4 and S5).

Our 4S-PQS analogues thus represent a promising novel scaffold that inhibits the labelling of PqsD by an active-site-directed probe in the lower micromolar range. We have previously described these compounds (**11**, **14**, and **16**) as potent inhibitors of the virulence factor elastase (LasB) of *Pseudomonas aeruginosa* [33]. However, the mechanism of inhibition was by direct binding to the active site of elastase. While elastolytic activity was completely inhibited with **11** even in situ we could not confirm any significantly large inhibition of rhamnolipid or pyocyanin production. Nevertheless, our new compounds may be useful scaffolds for the future development of a novel generation of PqsD inhibitors.

## Conclusion

Electrophilic probes represent powerful tools for investigating protein reactivity and discovering customized enzyme inhibitors. We discovered α-chloroacetamide probes selectively labelling the active site cysteine residue of the *Pseudomonas aeruginosa* quorum sensing signal synthase PqsD. While these findings may guide the future development of covalent PqsD inhibitors, we could also demonstrate the value of the probes as tools for investigating the reactivity of PqsD and apply them in a competitive screening approach. These led to the novel class of 4S-PQS analogues as potent in vitro inhibitors of the active-site labelling of PqsD. In combination, our probes and their inhibitors represent a valuable toolkit for investigating this important virulence-related enzyme.

## Supporting Information

File 1 Syntheses, and full compound characterization, experimental methods, and probe labelling.
