# Supplementary material for: Chemical probes for competitive profiling of the quorum sensing signal synthase PqsD of Pseudomonas aeruginosa
Source: Beilstein J Org Chem. 2016 Dec 20;12:2784–92. doi: 10.3762/bjoc.12.277 (PMC5238532; doi:10.3762/bjoc.12.277)
Supplement: File 1 — Syntheses, and full compound characterization, experimental methods, and probe labelling. [file Beilstein_J_Org_Chem-12-2784-s001.pdf]

Supporting Information

for

Chemical probes for competitive profiling of the quorum sensing signal synthase PqsD of *Pseudomonas aeruginosa*

Michaela Prothiwa, Dávid Szamosvári, Sandra Glasmacher and Thomas Böttcher*

Address: Department of Chemistry, Konstanz Research School Chemical Biology, University of Konstanz, 78457 Konstanz, Germany

Email: Thomas Böttcher - thomas.boettcher@uni-konstanz.de

* Corresponding author

Syntheses, and full compound characterization, experimental methods, and probe labelling

1. Synthesis

1.1. Materials and equipment

Chemical reagents and solvents were obtained from commercial suppliers (Sigma-Aldrich, Roth) and used without further purification. For thin-layer chromatography (TLC) silica gel was used and visualized by fluorescence quenching or by staining with phosphomolybdic acid or ninhydrin. For automated flash chromatography, a TELEDYNE ISCO CombiFlash® Rf (Lincoln, Nebraska, USA) was used with silica columns RediSep® Rf. Reversde-phase HPLC (RP-HPLC) was accomplished on an Spot Prep II Liquid Chromatography system (Armen Instrument, France). NMR spectra were obtained using a Bruker Avance III 400 equipped with a BBFO plus probe calibrated on the residual solvent peak. Mass spectra were recorded by ESI-TOF (Bruker Daltonics amicroTOFII) equipped with a Chromolith FastGaradient Rp18e 50*2 mm (Merck) column or ESI-IT (Bruker Daltonics Esquire 3000plus) equipped with a Nucleoshell 50*2 mm RP-18 2.7 µm (Macherey-Nagel).

1.2. Synthesis of activity-based probes

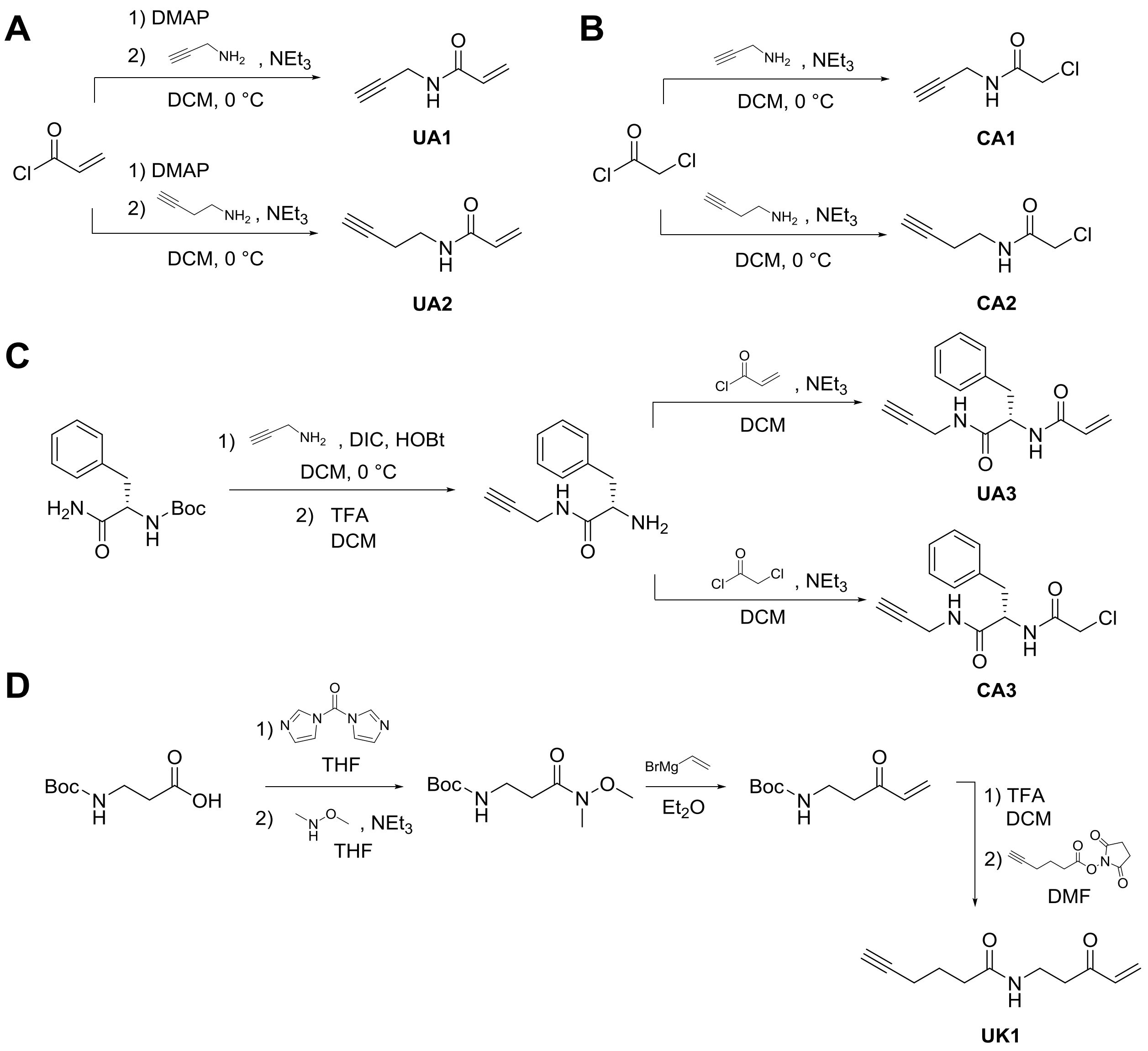

Figure S1: Synthetic scheme of ABPs. A) Synthesis of α,β-unsaturated amide probes **UA1–2**. B) Synthesis of α-chloroacetamide probes **CA1–2**. C) Synthesis of the phenylalanine-derived linker as common step for **UA3** and **CA3** synthesis. D) Synthesis of the α,β-unsaturated ketone probe **UK1**.

***N*-(Prop-2-yn-1-yl)acrylamide (UA1)[1]**

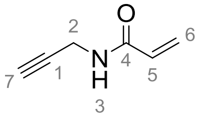

Acryloylchloride (1 mmol, 1 equiv, 81.5 µL) was preactivated with 4-dimethylaminopyridine (DMAP) (0.15 mmol, 0.15 equiv, 18 mg) in dry DCM (200 µL) under an argon atmosphere. The mixture was added dropwise to a precooled reaction mixture of propargylamine (1.5 mmol, 96 µL, 1.5 equiv) and triethylamine (1 mmol, 139 µL, 1 equiv) in dry DCM (500 µL) on ice under argon atmosphere. After stirring for 10 minutes on ice, the reaction mixture was warmed to room temperature and stirred overnight. The reaction mixture was diluted with DCM (25 mL), washed with $NaHCO_3$ (15 mL, 2×) and dried over anhydrous $MgSO_4$. The crude product in DCM was concentrated in vacuo and purified by column chromatography (Hex:EE, 2:1) obtaining a white solid (yield: 40%, 0.4 mmol, 44 mg). ^{1}H NMR (400 MHz, $CDCl_3$) δ 6.30 (dd, *J* = 17.0, 1.3 Hz, 1H, H-6_{cis}), 6.12 (dd, *J* = 17.0, 10.3 Hz, 1H, H-5), 5.67 (dd, *J* = 10.3, 1.4 Hz, 1H, H-6_{tans}), 4.12 (dd, *J* = 5.3, 2.6 Hz, 2H, H-2), 2.23 (t, *J* = 2.6 Hz, 1H, H-7). ^{13}C NMR (101 MHz, $CDCl_3$) δ 165.24, 130.14, 127.22, 79.31, 71.70, 29.26. $C_6H_7NO_2$: Exact mass: 109.0528. HRMS-ESI (MS+): $[M+Na]^+_{calc}$: m/z 132.0420. $[M+Na]^+_{found}$: m/z 132.0419 (Δppm: 0.8).

***N*-(But-3-yn-1-yl)acrylamide (UA2)[1]**

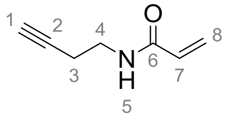

To a solution of acryloylchloride (1 mmol, 81.5 µL, 1 equiv) in dry DCM (200 µL) DMAP (0.15 mmol, 18 mg, 0.15 equiv) was added under argon atmosphere. The mixture was added dropwise to a precooled reaction mixture of 1-amino-3-butyne (1.5 mmol, 125 µL,1.5 equiv) and triethylamine (1 mmol, 139 µL, 1 equiv) in dry DCM (500 µL) at 0 °C under argon atmosphere. After stirring for 10 minutes on ice, the reaction mixture was warmed to room temperature and stirred overnight. The reaction mixture was diluted with DCM (25 mL), washed with $NaHCO_3$ (15 mL, 2×) and dried over anhydrous $MgSO_4$. The crude product in DCM was concentrated in vacuo and purified by column chromatography (Hex:EE, 2:1) to obtain a white solid (0.65 mmol, 80 mg, 65%). ^{1}H NMR (400 MHz, $CDCl_3$) δ 6.26 (dd, *J* = 17.0, 1.6 Hz, 1H, H-8_{cis}), 6.12 (dd, *J* = 17.0, 10.2 Hz, 1H, H-7), 5.62 (dd, *J* = 10.2, 1.6 Hz, 1H, H-8_{trans}), 3.46 (q, *J* = 6.4 Hz, 2H, H-4),

2.42 (td, J = 6.5, 2.7 Hz, 2H, H-3), 1.99 (t, J = 2.7 Hz, 1H, H-1). ^{13}C NMR (101 MHz, $CDCl_3$) δ 165.65, 130.83, 126.65, 81.54, 70.13, 38.19, 19.46. C_7H_9NO: Exact mass: 123.0684. HRMS-ESI (MS+): $[M+Na]^+_{calc}$: m/z 146.0576. $[M+Na]^+_{found}$: m/z 146.0573 (Δppm: 2.1).

2-Chloro-*N*-(prop-2-yn-1-yl)acetamide (CA1)[2]

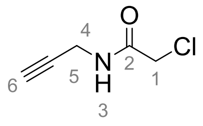

To a solution of propargylamine (1 mmol, 64 mL, 1 equiv) in dry DCM (2.5 mL) triethylamine (1.1 mmol, 153 µL, 1.1. equiv) was added under argon atmosphere. The stirring solution was cooled to 0 °C and chloroacetyl chloride (1.2 mmol, 95 µL, 1.2 equiv) was added under argon atmosphere. The solution was stirred for 60 minutes at 0 °C. The reaction mixture was warmed to rt and stirred for additional two hours. Afterwards, water (5 mL) was added and extracted with DCM (5 mL, 3×). The combined organic layers were washed with 10% HCl and brine. After drying over anhydrous $MgSO_4$ the solvent was removed under reduced pressure and the product was purified by C_{18} RP-HPLC (5% MeCN in MilliQ water to 95% MeCN in MilliQ water in 35 minutes. Flow-rate: 15 mL/min) to obtain the product (0.7 mmol, 92 mg, 70%). ^{1}H NMR (400 MHz, $CDCl_3$) δ 6.85 (s, 1H. NH), 4.08 (q, J = 5.45, 2.60, 2H, H-4), 4.05 (s, 2H, H-1), 2.27 (s, 1H, H-6). ^{13}C NMR (101 MHz, $CDCl_3$) δ 165.75, 78.67, 72.36, 42.50, 29.73. C_5H_6ClNO: Exact mass: 131.0138. HRMS-ESI (MS+): $[M+Na]^+_{calc}$: m/z 154.0030. $[M+Na]^+_{found}$: m/z 154.0029 (Δppm: 0.7).

***N*-(But-3-yn-1-yl)-2-chloroacetamide (CA2)[2]**

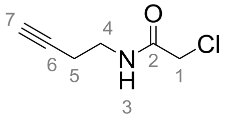

To a solution of 1-amino-3-butyne (1 mmol, 82 mL,1 equiv) in dry DCM (2.5 mL) triethylamine (1.1 mmol, 153 µL, 1.1. equiv) was added under argon atmosphere. The stirring solution was cooled to 0 °C. Chloroacetyl chloride (1.2 mmol, 95 µL, 1.2 equiv) was added to the precooled solution under argon atmosphere and stirred for 60 minutes at 0 °C. The reaction mixture was warmed to rt and stirred for additional two hours. Afterwards, water (5 mL) was added and extracted with DCM (5 mL, 3×). The combined organic layers were washed with 10% HCl and brine. After drying over anhydrous $MgSO_4$ the solvent was removed under reduced pressure and the product was purified by C_{18} RP-HPLC (5% MeCN in MilliQ water to 95% MeCN in MilliQ

water in 35 minutes. Flow-rate: 15mL/min) to obtain a white solid (0.8 mmol, 126 mg, 86%). ^{1}H NMR (400 MHz, $CDCl_3$) δ 7.00 (s, 1H, NH), 3.99 (s, 2H, H-1), 3.39 (q, *J* = 6.4 Hz, 2H, H-4), 2.38 (dt, *J* = 6.6, 2.7 Hz, 2H, H-5), 2.00 (t, *J* = 2.7 Hz, 1H, H-7). ^{13}C NMR (101 MHz, $CDCl_3$) δ 166.11, 80.90, 70.36, 42.56, 38.29, 19.12. C_6H_8ClNO: Exact mass: 145.0294. HRMS-ESI (MS+): $[M+Na]^+_{calc}$: m/z 168.0186. $[M+Na]^+_{found}$: m/z 168.0166 (Δppm: 11.9).

N-tert-butyloxycarbonyl-L-phenylalanyl-propargylamide

.

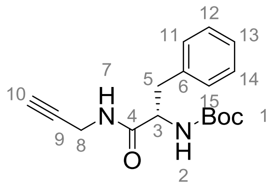

To a stirred solution of *N*-*tert*-butyloxycarbonyl-L-phenylalanine (2 mmol, 531 mg, 1 equiv) and HOBt (2 mmol, 310 mg, 1 equiv) in dry DCM (20 mL), DIC (2 mmol, 310 µL, 1 equiv) was added under argon atmosphere at 0 °C and stirred for 5 minutes. Afterwards propargylamine (4.8 mmol, 200 µL, 2.4 equiv) was added at 0 °C. The resulting yellow-orange solution was stirred for 20 hours at rt. The reaction mixture was concentrated under reduced pressure, extracted with ethyl acetate (40 mL), washed with water (25 mL, 4×) and reduced in vacuo. The residual oil was purified by flash chromatography (Hex:EE, 7: 3) to obtain a yellow-white solid (0.9 mmol, 540 mg, 90%). ^{1}H NMR (400 MHz, $CDCl_3$) δ 7.37 – 7.22 (m, 5H, H-11-15), 6.41 (s, 1H, H-2), 5.18 (s, 1H, H-7), 4.41 (s, 1H, H-3), 4.03 (dt, *J* = 5.9, 2.8 Hz, 2H, H-8), 3.11 (t, *J* = 7.6 Hz, 2H, H-5), 2.24 (t, *J* = 2.6 Hz, 1H, H-10), 1.45 (s, 9H, Boc). ^{13}C NMR (101 MHz, $CDCl_3$) δ 171.21, 155.53, 136.67, 129.45, 128.76, 127.05, 80.28, 79.22, 71.70, 55.84, 38.66, 29.18, 28.38.

L-Phenylalanylpropargylamide

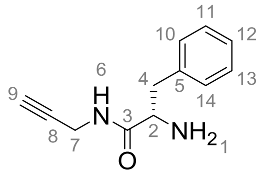

N-*tert*-butyloxycarbonyl-L-phenylalanylpropargylamide (0.9 mmol, 540 mg) was dissolved in TFA/DCM (1:1, 4 mL) and stirred for 5 hours at rt. The clear solution turned yellow. To neutralize the solution, aqueous ammonia solution (28% NH_3 in H_2O, 5 mL) was added. Afterwards water (10 mL) was added and the solution was extracted with 150 mL ethyl acetate. The combined

organic layers were dried over anhydrous $MgSO_4$ and the solvent was evaporated in vacuo. The product was co-evaporated with chloroform/toluene (5×) and the crude product was directly used for the following reaction without further purification. ^{1}H NMR (400 MHz, CD_3OD) δ 8.74 (s, 2H, H-1), 7.36 – 7.21 (m, 5H, H-10-14), 4.11 – 4.01 (m, 1H, H-2), 4.00 – 3.85 (m, 2H, H-7), 3.21 – 3.01 (m, 2H, H-4), 2.55 (t, *J* = 2.5 Hz, 1H, H-9). ^{13}C NMR (101 MHz, CD_3OD) δ 170.81, 135.90, 130.54, 129.81, 128.49, 79.84, 72.67, 55.98, 39.14, 29.59.

(S)-*N*-(1-Oxo-3-phenyl-1-(prop-2-yn-1-ylamino)propan-2-yl)acrylamide (UA3)[1]

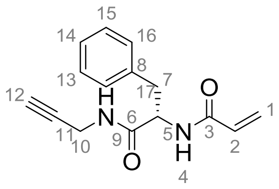

A solution of acryloylchloride (1 mmol, 81.5 µL, 1 equiv) in dry DCM (300 µL) was added dropwise to a mixture of L-phenylalanylpropargylamide (1.5 mmol, 203 mg, 1.5 equiv) and triethylamine (2 mmol, 280 µL, 2 equiv) in dry DCM (700 µL) at 0 °C under argon atmosphere. After stirring for 10 minutes at 0 °C, the mixture was warmed to rt and stirred overnight. Afterwards the mixture was diluted with DCM (25 mL), washed two times with $NaHCO_3$ (15 mL), dried over anhydrous $MgSO_4$ and concentrated in vacuo. The product was purified by flash chromatography (Hex:EE, 2:1) to obtain a yellow solid (50%, 0.5 mmol, 126 mg). ^{1}H NMR (400 MHz, CD_3OD) δ 7.35 – 7.22 (m, 5H, H-13-17), 6.32 (dd, *J* = 17.1, 10.0 Hz, 1H, H-2), 6.22 (dd, *J* = 17.1, 2.0 Hz, 1H, H-1_{cis}), 5.69 (dd, *J* = 10.0, 2.0 Hz, 1H, H-1_{tans}), 4.72 (dd, *J* = 8.4, 6.3 Hz, 1H, H-5), 4.03 – 3.91 (dd, 2H, H-10), 3.19 (dd, *J* = 13.7, 6.3 Hz, 1H, H-7), 2.98 (dd, *J* = 13.8, 8.4 Hz, 1H, H-7), 2.61 (t, *J* = 2.6 Hz, 1H, H-12). ^{13}C NMR (101 MHz, CD_3OD) δ 173.10, 167.80, 138.26, 131.64, 130.33, 129.47, 127.82, 127.26, 80.29, 72.31, 56.09, 39.14, 29.52. $C_{15}H_{16}N_2O_2$: Exact mass: 256.1212. HRMS-ESI (MS+): $[M+Na]^+_{calc}$: m/z 279.1104. $[M+Na]^+_{found}$: m/z 279.1085 (Δppm: 6.8).

(S)-2-(2-Chloroacetamido)-3-phenyl-*N*-(prop-2-yn-1-yl)propanamide (CA3)[2]

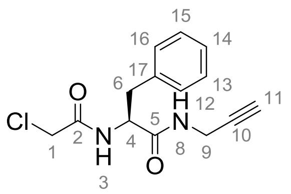

To a solution of L-phenylalanylpropargylamide (1 mmol, 202 mg, 1 equiv) in dry DCM (2.5 mL) triethylamine (1.1 mmol, 300 µL, 2 equiv) was added under argon atmosphere. The stirring solution was cooled to 0 °C and chloroacetyl chloride (1.2 mmol, 95 µL, 1.2 equiv) was added under argon and stirred for 60 minutes at 0 °C. Afterwards the reaction mixture was stirred at rt for 2 h. Water (10 mL) was added and the solution was extracted with DCM (10 mL, 3×). The combined organic layers were washed with 10% HCl and brine, dried over anhydrous $MgSO_4$ and concentrated in vacuo. The product was precipitated in DCM forming a yellow-orange solid (0.11 mmol, 30 mg, 11%). ^{1}H NMR (400 MHz, DMSO-d_6) δ 8.56 (t, *J* = 5.5 Hz, 1H, H-8), 8.42 (d, *J* = 8.4 Hz, 1H, H-3), 7.22 (m, *J* = 19.3, 6.5, 5.5 Hz, 5H, H-12-16), 4.50 (td, *J* = 8.9, 4.9 Hz, 1H, H-4), 4.03 (d, *J* = 3.0 Hz, 2H, H-1), 3.87 (dt, *J* = 5.9, 3.0 Hz, 2H, H-9), 3.31 (s, 2H, H-11), 3.12 (t, *J* = 2.6 Hz, 1H, H-11), 2.99 (dd, *J* = 13.7, 4.9 Hz, 1H, H-6), 2.79 (dd, *J* = 13.7, 9.2 Hz, 1H, H-10). ^{13}C NMR (101 MHz, DMSO) δ 170.27, 165.56, 137.32, 129.12, 128.04, 126.33, 80.74, 73.15, 54.02, 42.42, 37.62, 27.96. $C_{14}H_{15}ClN_2O_2$: Exact mass: 278.0822. HRMS-ESI (MS+): $[M+Na]^+_{calc}$: m/z 301.0714 $[M+Na]^+_{found}$: m/z 301.0669 (Δppm: 15).

***tert*-Butyl (3-(methoxy(methyl)amino)-3-oxopropyl)carbamate**

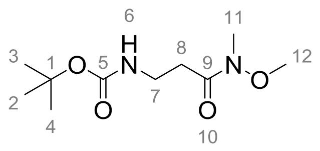

Boc-β-alanine (5.3 mmol, 1 g, 1 equiv) was dissolved in dry THF (40 mL) and carbonyldiimidazole (5.8 mmol, 0.82 g, 1.1 equiv) was added under argon atmosphere. The reaction mixture was stirred at rt for 4 h. In a separate flask *N,O*-dimethylhydroxylamine hydrochloride (5.8 mmol, 0.57 g, 1.1 equiv) and triethylamine (11.6 mmol, 1.6 mL, 2.2 equiv) was added to dry THF (15 mL) under argon atmosphere and stirred for 4 h at rt. The first

reaction mixture was slowly added to the second one under argon atmosphere, and the white suspension was stirred over night at rt. The white solid was filtered and washed with THF. The filtrate was concentrated under reduced pressure to yield a yellow oil. The oil was dissolved in DCM (100 mL), washed with 0.1 M HCl (3 × 150 mL) and water (150 mL). The combined organic layers were dried over anhydrous $MgSO_4$ and the solvent was removed in vacuo to obtain a white-yellow solid (3.95 mmol, 0.92 g, 75%). 1H NMR (400 MHz, $CDCl_3$) δ 5.21 (s, 1H, H-6), 3.67 (s, 3H, H-12), 3.42 (q, *J* = 6.0 Hz, 2H, H-7), 3.18 (s, 3H, H-11), 2.63 (t, *J* = 5.8 Hz, 2H, H-8), 1.43 (s, 9H, H-2-4). 13C NMR (101 MHz, $CDCl_3$) δ 173.23 156.23, 79.34, 61.48, 36.08, 32.56, 32,04, 28.68. $C_{10}H_{20}N_2O_4$: Exact mass: 232.14. MS-ESI (MS^+). $[M+Na]^+_{calc}$: m/z 255.13. $[M+Na]^+_{found}$: m/z 255.10 (Δppm: 117).

tert-Butyl (3-oxopent-4-en-1-yl)carbamate

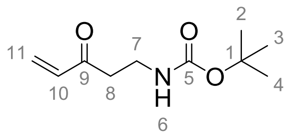

tert-Butyl (3-(methoxy(methyl)amino)-3-oxopropyl)carbamate (2.2 mmol, 0.51 g, 1 equiv) in dry diethyl ether (15 mL) was cooled to 0 °C under argon atmosphere. Within three minutes vinylmagnesium bromide (1 M solution in THF, 11 mmol, 15 mL, 5 equiv) was added dropwise to the suspension and stirred for 90 min at 0 °C. The reaction was quenched with cold 1 M HCl (24 mL) and extracted with cold diethylether (3 × 12 mL). The combined organic layers were washed with saturated sodium hydrogen carbonate solution and dried over anhydrous magnesium sulfate. After removing the solvent in vacuo the product was obtained as yellow oil (0.96 mmol, 0.193 g, 43%). ^{1}H NMR (400 MHz, $CDCl_3$) δ 6.35 (dd, *J* = 17.7, 10.3 Hz, 1H, H-10), 6.24 (dd, *J* = 17.8, 1.3 Hz, 1H, $H\text{-}11_{cis}$), 5.89 (dd, *J* = 10.3, 1.3 Hz, 1H, $H\text{-}11_{trans}$), 5.02 (s, 1H, H-6), 3.43 (q, *J* = 6.0 Hz, 2H, H-7), 2.83 (t, *J* = 5.8 Hz, 2H ,H-8), 1.43 (s, 9H, H-2-4). ^{13}C NMR (101 MHz, $CDCl_3$) δ 156.09, 136.66, 129.02, 79.19, 35.94, 32.41, 28.53. $C_{10}H_{17}NO_3$: Exact mass: 199.12. MS-ESI (MS+): $[M+Na]^+_{calc}$: m/z 222.11. $[M+Na]^+_{found}$: m/z 222.11.

5-Aminopent-1-en-3-one

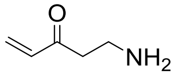

tert-Butyl (3-oxopent-4-en-1-yl)carbamate (0.96 mmol, 0.193 g) was deprotected by stirring in DCM/TFA (1:1, v/v, 5 mL in total) at rt overnight. The product was co-evaporated with DCM (4×) and toluene (4×) and directly used in the next experiment without further purification.

***N*-(3-Oxopent-4-en-1-yl)hex-5-ynamide (UK1)**

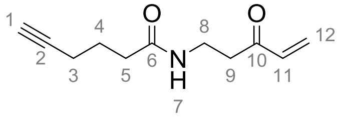

To a stirring solution of 5-aminopent-1-en-3-one (0.96 mmol, 1 equiv) and triethylamine (660 µL) in DMF (4.5 mL) 2,5-dioxopyrrolidin-1-yl hex-5-ynoate (1.1 mmol, 0.23 g, 1.1 equiv) was added slowly and the reaction mixture was stirred overnight at rt. The solvent was removed under reduced pressure and the crude product was co-evaporated with chloroform (5 × 5 mL). The product was purified by C_{18} RP-HPLC (5% MeCN in MilliQ water to 95% in MilliQ water. Flow-rate 15 mL/min) to obtain a yellow solid (0.022 g, 0.11 mmol, 11%). ^{1}H NMR (400 MHz, $CDCl_3$) δ 6.31 (dd, *J* = 17.7, 10.3 Hz, 1H, H-11), 6.21 (dd, *J* = 17.8, 1.2 Hz, 1H, H-12_{cis}), 6.14 (s, 1H, H-7), 5.87 (dd, *J* = 10.3, 1.1 Hz, 1H, H-12_{trans}), 3.51 (q, *J* = 5.9 Hz, 2H, H-8), 2.82 (t, *J* = 5.7 Hz, 2H, H-9), , 2.28 – 2.22 (dt, 2H, H-5), 2.19 (dt, *J* = 6.9, 2.7 Hz, 2H, H-3), 1.93 (dt, *J* = 5.4, 2.6 Hz, 1H, H-1), 1.84 – 1.75 (m, 2H, H-4). ^{13}C NMR (101 MHz, $CDCl_3$) δ 200.32, 172.53, 136.52, 129.38, 83.53, 69.31, 38.96, 35.18, 32.54, 24.24, 17.96. $C_{11}H_{15}NO_2$: Exact mass: 193.1103. HRMS-ESI (MS+): $[M+Na]^+_{calc}$: m/z 216.0995. $[M+Na]^+_{found}$: m/z 216.0986 (Δppm: 4.2).

4-(Benzyloxy)-2-heptylquinoline (3a)

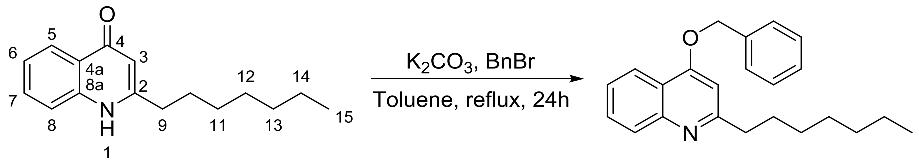

To a solution of 0.253 g (1.04 mmol) 2-heptylquinolin-4-one (**1**) and 144 mg K_2CO_3 (1.04 mmol) in 5 mL dry toluene was added dropwise 150 µL (1.25 mmol) benzyl bromide and the reaction was stirred at reflux temperatures for 24 h. The reaction was allowed to cool to room temperature and was poured into water and extracted with ethyl acetate and the combined organic phases washed with brine, dried with $MgSO_4$, filtered and evaporated. The residue was purified by column chromatography on silica gel using DCM to DCM/MeOH (98.5:1.5). The product was received as yellow oil (233 mg, 67%). ^{1}H-NMR ($CDCl_3$ 399.79 MHz) δ (ppm): 0.89 (m, 3H, H-15), 1.20-1.40 (m, 8H, H-11-14), 1.76 (m, 2H, H-10), 2.88 (m, 2H, H-9), 5.37 (s, 2H, H-16), 6.94 (s, 1H, H-3), 7.36 (m, 1H, H-4´), 7.42 (m, 2H, H-3´), 7.47 (ddd, *J* = 8.7 Hz, *J* = 8.4 Hz, *J* = 1.0 Hz, 1H, H-6), 7.53 (m, 2H, H-2´), 7.69 (ddd, *J* = 8.7 Hz, *J* = 8.4 Hz, *J* = 1.4 Hz, 1H, H-7), 7.90 (d, *J* = 8.4 Hz, 1H, H-8), 8.19 (dd, *J* = 8.4 Hz, *J* = 1.1 Hz, 1H, H-5). ^{13}C-NMR ($CDCl_3$ 100.53 MHz) δ (ppm): 14.4 (C-15), 23.7, 30.3, 30.5, 32.9 (C-11-14), 31.3 (C-10), 40.1 (C-9), 71.6 (C-16) 102.7 (C-3), 121.4 (C-4a), 122.9 (C-5), 126.3 (C-6), 127.9 (C-8), 128.7 (C-2´), 129.3 (C-4´), 129.7 (C-3´), 131.2 (C-7), 137.5 (C-1´), 149.5 (C-8a), 163.3 (C-4), 166.0 (C-2). HR-ESI-MS: m/z = 334.2251 $[M+H]^+$, calc. for $C_{23}H_{28}NO^+$ = 334.2171, m/z = 667.4290 $[2M+H]^+$, calc. for $C_{46}H_{55}N_2O_2^+$ = 667.4264.

2-Heptylquinolin-4-one oxime (3)

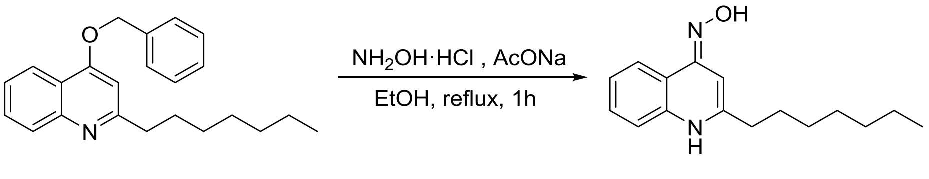

A solution of 0.132 g (0.396 mmol) 4-(benzyloxy)-2-heptylquinoline (**3a**), 1.5 g (0.02 mol) $NH_2OH{\cdot}HCl$ and 1.8 g (0.02 mol) sodium acetate in 40 mL EtOH was stirred at reflux conditions for 1 h. After the reaction cooled to room temperature the solids were filtered off and washed with EtOH. The filtrates and washings were combined and evaporated under reduced pressure. The residue was dissolved in DCM and washed two times with water. By dewatering the organic phase with brine, a colorless foam between the DCM and brine appeared. The brine phase and

the foam were extracted three times with DCM. The organic phases were discarded. The brine phase was basified with 1 N NaOH whereupon the foam disappeared. The brine phase was extracted two times with ethyl acetate. The combined organic phases were tried with $MgSO_4$, filtered and evaporated leaving a yellow crystalline solid which was purified by column chromatography on silica gel using DCM. The product was received as yellow crystals (50 mg, 49%). ^{1}H-NMR (DMSO-d_6 600.33 MHz) δ (ppm): 0.85 (m, 3H, H-15), 1.20-1.36 (m, 8H, H-11-14), 1.59 (m, 2H, H-10), 2.33 (m, 2H, H-9), 5.92 (s, 1H, H-3), 6.98 (dd, *J* = 8.5 Hz, *J* = 8.1 Hz, 1H, H-6), 7.10 (d, *J* = 8.2 Hz, 1H, H-8), 7.28 (ddd, *J* = 8.5 Hz, *J* = 8.2 Hz, *J* = 1.2 Hz, 1H, H-7), 7.79 (dd, *J* = 8.1 Hz, *J* = 1.2 Hz, 1H, H-5), 9.54 (s, 1H, =N-OH), 9.74 (s, br, 1H, H-1). ^{13}C-NMR (DMSO-d_6 150.95 MHz) δ (ppm): 14.0 (C-15), 28.5 (C-10), 22.1, 28.0, 28.6, 31.2 (C-11-14), 33.5 (C-9), 92.4 (C-3), 116.6 (C-8), 118.7 (C-4a), 121.7 (C-6), 122.3 (C-5), 128.8 (C-7), 138.3 (C-8a), 146.0 (C-2), 147.1 (C-4). HR-ESI-MS: m/z = 259.1874 $[M+H]^+$, calc. for $C_{16}H_{23}N_2O^+$ = 259.1810, m/z = 539.3409 $[2M+Na]^+$, calc. for $C_{32}H_{44}N_4NaO_2^+$ = 539.3362.

2-Heptyl-chromen-4-one, 1-O-HHQ (4)

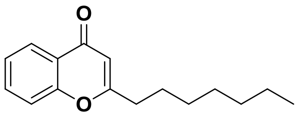

To a solution of 2 g of 2´-hydroxyacetophenone (14.7 mmol) and 3.3 g *t*-BuOK (29.4 mmol, 2 eq.) in 50 mL THF was added 2.51 mL octanoyl chloride (14.7 mmol) and the mixture stirred at 80 °C for 4 h. The reaction was allowed to cool to room temperature and 10 mL conc. H_2SO_4 was added dropwise while cooling the reaction in a water bath. The mixture was heated to 90 °C and kept at this temperature for 90 min. The reaction was allowed to cool to room temperature and was poured on ice. The aqueous phase was extracted with ether (3 × 100 mL) and the combined organic phases were washed once with water and dried with $MgSO_4$. After filtration, the solvent was evaporated and the residue purified by column chromatography on silica 60 using ethyl acetate/petrol ether 9:1. The product was received as yellow oil (2.164 g, 60.2%).^{1}H-NMR ($CDCl_3$ 400.13 MHz) δ (ppm): 0.88 (m, 3H, H-15), 1.22-1.45 (m, 8H, H-11-14), 1.74 (m, 2H, H-10), 2.61 (t, *J* = 7.6 Hz, 2H, H-9), 6.18 (s, 1H, H-3), 7.37 (m, 1H, H-6), 7.42 (d, *J* = 8.4 Hz, 1H, H-8), 7.63 (m, 1H, H-7), 8.18 (dd, *J* = 8.0 Hz, *J* = 1.6 Hz, 1H, H-5). ^{13}C-NMR ($CDCl_3$ 100.26 MHz) δ (ppm): 14.2 (C-15), 22.7, 29.06, 29.09, 31.8, (C-11-14), 26.9 (C-10), 34.5 (C-9), 109.9

(C-3), 118.0 (C-8), 123.9 (C-4a), 125.0 (C-6), 125.8 (C-5), 133.5 (C-7), 156.7 (C-8a), 170.0 (C-2), 178.6 (C-4). ESI-MS: m/z = 244.90 $[M+H]^+$, calc. for $C_{16}H_{21}O_3^+$ = 245.15, m/z = 489.15 $[2M+H]^+$, calc. for $C_{32}H_{41}O_4^+$ = 489.30.

2-Heptyl-chromene-4-thione (5)

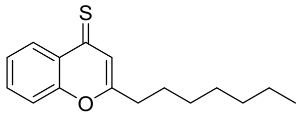

A mixture of 250 mg (1.023 mmol) of 2-heptyl-4*H*-chromen-4-one (**4**) and P_4S_{10} (342 mg, 1.53 mmol) in 10 mL pyridine was refluxed for 4 h. The mixture was allowed to cool to room temperature and poured into ice cold water. The solution was acidified with 6 M HCl and extracted two times with DCM. The combined organic phases were dried over $MgSO_4$ and evaporated. The brown oil was purified using silica gel chromatography in petrol ether/ethyl acetate 1:4. The product was obtained as a dark red oil (201 mg, 75%). *Rf* = 0.95 (PE/EA 1:4). ^{1}H-NMR ($CDCl_3$ 399.79 MHz) δ (ppm): 0.89 (m, 3H, H-15), 1.25 – 1.44 (m, 8H, H-11, H-12, H-13, H-14), 1.77 (m, 2H, H-10), 2.59 (t, *J* = 7.6 Hz, 2H, H-9), 7.16 (s, 1H, H-3), 7.39 (ddd, 1H, *J* = 8.4 Hz, *J* = 8.1 Hz, *J* = 1.1 Hz, H-6), 7.43 (dd, 1H, *J* = 8.4 Hz, *J* = 1.1 Hz, H-8), 7.67 (ddd, 1H, *J* = 8.4 Hz, *J* = 8.1 Hz, *J* = 1.6 Hz, H-7), 8.58 (dd, 1H, *J* = 8.1 Hz, *J* = 1.6 Hz, H-5). ^{13}C-NMR ($CDCl_3$ 100.53 MHz) δ (ppm): 14.2 (C-15), 22.7, 29.1, 29.2, 31.8 (C-11-14), 27.0 (C-10), 33.9 (C-9), 118.4 (C-8), 123.0 (C-3), 126.1 (C-6), 128.9 (C-5), 129.8 (C-4a), 134.0 (C-7), 152.1 (C-8a), 161.2 (C-2), 202.9 (C-4). HR-ESI-MS: m/z = 261.1342 $[M+H]^+$, calc. for $C_{16}H_{21}OS^+$ = 261.1313, m/z = 521.2604 $[2M+H]^+$, calc. for $C_{32}H_{41}O_2S_2^+$ = 521.2548.

2-Heptyl-thiochromene-4-thione (7)

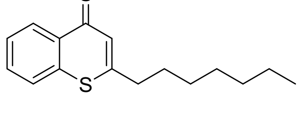

A mixture of 65.6 mg (0.252 mmol) of 2-heptyl-4*H*-thiochromen-4-one (**6**) and P_4S_{10} (84 mg, 0.378 mmol) in 10 mL pyridine was refluxed for 4 h. The mixture was allowed to cool to room

temperature and poured into ice cold water. The solution was acidified with 6 M HCl and extracted two times with DCM. The combined organic phases were dried over $MgSO_4$ and evaporated. The brown oil was purified using silica gel chromatography in petrol ether/ethyl acetate 1:4. The product was obtained as a dark brown oil (47 mg, 67%). *Rf* = 0.9 (PE/EA 1:4). ^{1}H-NMR (DMSOd$_6$ 399.79 MHz) δ (ppm): 0.85 (m, 3H, H-15), 1.20 – 1.40 (m, 8H, H-11, H-12, H-13, H-14), 1.70 (m, 2H, H-10), 2.76 (t, *J* = 7.6 Hz, 2H, H-9), 7.66 (m, 1H, H-6), 7.78 (m, 1H, H-7), 7.94 (d, *J* = 8.2 Hz, 1H, H-8), 7.96 (s, 1H, H-3), 8.87 (dd, 1H, *J* = 8.4 Hz, *J* = 1.3 Hz, H-5). ^{13}C-NMR (DMSO-d$_6$ 100.53 MHz) δ (ppm): 13.8 (C-15), 22.0, 28.18, 28.21, 31.0 (C-11-14), 29.5 (C-10), 36.0 (C-9), 127.5 (C-8), 129.1 (C-6), 131.1 (C-5), 131.9 (C-7), 133.1 (C-8a), 136.0 (C-4a), 136.4 (C-3), 148.9 (C-2), 203.2 (C-4). HR-ESI-MS: m/z = 277.1283 $[M+H]^+$, calc. for $C_{16}H_{21}S_2^+$ = 277.1085, m/z = 553.2477 $[2M+H]^+$, calc. for $C_{32}H_{41}S_4^+$ = 553.2091.

2-Heptylchroman-4-one (8)

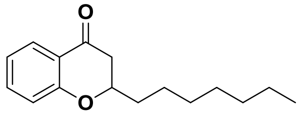

To a mixture of 0.733 g (3.00 mmol) 2-heptyl-chromen-4-one (**4**) and 150 mg 10% Pd/C in 15 mL dry MeOH was added 570 mg (9.00 mmol) ammonium formate and the reaction mixture refluxed for 3 h. After filtration and evaporation of the solvent, the residue was suspended in ether and washed twice with water and once with brine, dried with $MgSO_4$, filtered and evaporated. The residue was purified by column chromatography on silica gel using dichloromethane. The product was received as a colorless oil (562 mg, 76%). *Rf* = 0.9 (DCM). Unreacted starting material was recovered by elution with 10% MeOH in DCM (170 mg, 23%). ^{1}H-NMR ($CDCl_3$ 399.79 MHz) δ (ppm): 0.89 (m, 3H, H-15), 1.24-1.39 (m, 8H, H-11-14), 1.46 (m, 1H, H-10), 1.53 (m, 1H, H-10), 1.70 (m, 1H, H-9), 1.88 (m, 1H, H-9), 2.68 (d, *J* = 8.0 Hz, 2H, H-3), 4.43 (m, 1H, H-2), 6.97 (m, 1H, H-8), 7.00 (m, 1H, H-6), 7.46 (m, 1H, H-7), 7.87 (dd, *J* = 7.9 Hz, *J* = 1.8 Hz, 1H, H-5). ^{13}C-NMR ($CDCl_3$ 100.53 MHz) δ (ppm): 14.3 (C-15), 22.8, 29.3, 29.5, 31.9 (C-11-14), 25.1 (C-10), 35.3 (C-9), 43.2 (C-3), 78.1 (C-2), 118.2 (C-8), 121.2 (C-4a), 121.3 (C-6), 127.2 (C-5), 136.2 (C-7), 161.9 (C-8a), 192.8 (C-4). ESI-MS: m/z = 269.1 $[M+H]^+$, calc. for $C_{16}H_{22}O_2 + H^+$ = 269.2.

2-Heptylthiochroman-4-one (9)

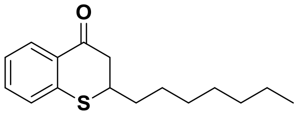

To a solution of 504 mg trans-2-decenic acid (2.96 mmol) in 1.5 mL dry DCM in a Schlenck tube with a septum under nitrogen was added 0.3 mL thiophenol (2.94 mmol) and the mixture was cooled with an ice-bath. Under nitrogen 3 mL trifluoromethanesulfonic acid was added and the mixture stirred at 0 °C for 5 min. After that, the mixture was heated in an oil bath at 130 °C with a reflux cooler for 5 min. the reaction was allowed to cool to room temperature and was poured on ice. DCM was added and the phases separated. The aqueous phase was washed with DCM and the combined organic phases were washed twice with sat. $NaHCO_3$ sol. and brine. The organic phases were dried with $MgSO_4$, filtered and the solvent evaporated. The product was purified by column chromatography on silica 60 with PE/DCM 4:1 and PE/DCM 1:1. The product was collected at last as the second fluorescent substance on TLC. The product was obtained as slightly yellow oil (m = 413 mg, 53.2%). *Rf* = 0.55 (PE/DCM 1:1). ^{1}H-NMR ($CDCl_3$ 399.79 MHz) δ (ppm): 0.88 (m, 3H, H-15), 1.20-1.37 (m, 8H, H-11-14), 1.46 (m, 2H, H-10), 1.71 (m, 2H, H-9), 2.80 (dd, *J* = 16.2 Hz, *J* = 11.1 Hz, 1H, H-3), 3.04 (dd, *J* = 16.2 Hz, *J* = 3.1 Hz, 1H, H-3), 3.50 (m, 1H, H-2), 7.16 (m, 1H, H-6), 7.27 (m, 1H, H-8), 7.38 (m, 1H, H-7), 8.08 (dd, *J* = 8.0 Hz, *J* = 1.5 Hz, 1H, H-5). ^{13}C-NMR ($CDCl_3$ 100.53 MHz) δ (ppm): 14.2 (C-15), 22.8, 29.2, 29.3, 31.9 (C-11-14), 26.8 (C-10), 34.7 (C-9), 41.8 (C-2), 46.4 (C-2), 125.0 (C-6), 127.8 (C-8), 129.0 (C-5), 130.8 (C-4a), 133.6 (C-7), 141.9 (C-8a), 194.9 (C-4). EI-MS: m/z = 262 [M], calc. for $C_{16}H_{22}OS$• = 262; m/z = 136 [M - C_9H_{18}], calc. for C_7H_4OS• = 136; m/z = 163 [M - C_7H_{15}], calc. for C_9H_7OS• = 163.

2-Heptyl-3-hydroxy-chromen-4-one (13)

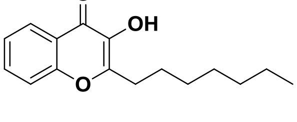

To a solution of 860 mg 2-heptylchroman-4-one (**8**)(3.52 mmol) in 50 mL toluene were added 1.2 mL *t*-BuOOH and 1.2 mL Triton B at 0 °C. After stirring at 0 °C for 5 min the reaction was continued for 30 min at room temperature. Additional 1.2 mL *t*-BuOOH and 1.2 mL Triton B were

added and the mixture stirred for another 30 min at room temperature. The reaction was diluted with ethyl acetate and washed with water and brine, dried with $MgSO_4$, filtered and the solvent evaporated. The crude residue was dissolved in 30 mL DCM and 810 mg TsOH monohydrate was added. The mixture was stirred at room temperature for 2 h. The solvent was evaporated and the residue purified by column chromatography on silica 60 using ethyl acetate/ petrol ether 5:1. The product was received as yellow oil which crystallized (380 mg, 41.5%).^{1}H-NMR ($CDCl_3$ 400.13 MHz) δ (ppm): 0.88 (t, *J* = 7.0 Hz, 3H, H-15), 1.22 – 1.47 (m, 8H, H-11-14), 1.77 (quint., *J* = 7.5 Hz, 2H, H-10), 2.84 (t, *J* = 7.7 Hz, 2H, H-9), 6.23 (s, 1H, -OH), 7.37 (t, *J* = 7.3 Hz, 1H, H-6), 7.47 (d, *J* = 8.5 Hz, 1H, H-8), 7.64 (t, *J* = 7.7 Hz, 1H, H-7), 8.22 (d, *J* = 7.8 Hz, 1H, H-5). 13C-NMR (CDCl3 100.61 MHz) δ (ppm): 14.2 (C-15), 22.7 (C-14), 26.8 (C-10), 29.1 (C-9), 29.1 (C-12), 29.3 (C-11), 31.8 (C-13), 118.3 (C-8), 121.6 (C-4a), 124.4 (C-6), 125.6 (C-5), 133.1 (C-7), 138.3 (C-3), 152.6 (C-2), 155.8 (C-8a), 172.6 (C-4). ESI-MS: m/z = 260.9 $[M+H]^+$, calc. for $C_{16}H_{20}O_3 + H^+$ = 261.2.

2. Biochemical methods

2.1. Materials and equipment

Overnight cultures and general bacteria liquid cultures were incubated in an Ecotron shaking incubator of INFORS HT (Einsbach, Germany). The optical density of cells at a wavelength of λ = 600 nm (OD_{600}) was measured with a photometer of Eppendorf (BioPhotometer® plus). The PeqLab System was used for the preparation of SDS Pages. The analysis of the gels was conducted with a Fusion-FX7 Advanced of Vilber Lourmat (Eberhardzell, Germany) and the software CaptAdvance. Fluorescence of SDS gels was recorded with a FUSION-FX7 Advance with EPI-UV/Blue and SUPER-BRIGHT imaging system equipped with a 4.2/10 Mio Pixel CCD-Camera, Fusion SPECTRA LED EPI Illuminator Device for excitation of fluorescent dyes and a F595 Y3 camera filter for emission maxima > 550 nm (VWR, Erlangen, Germany) The primers were synthesized by Metabion International AG and were delivered in 100 µM stocks.

2.2. Recombinant expression and mutagenesis

Pseudomonas aeruginosa PAO1 strain ATCC 15692 (DSMZ 22644) was grown in LB-(lysogenic broth)-Lennox medium at 37 °C overnight and the genome extracted using a Puregene Yeast/Bacteria Kit B (Qiagen).

For cloning, the Gateway® Technology was used. The attB1 forward primer and the attB2 reverse primer were designed to yield attB-PCR products. To produce an N-terminal strep-tagged protein, the DNA sequence for a Strep-tag was included in the pDest007 plasmid. The pqsD sequence was amplified by a Phusion High-Fidelity PCR kit (ThermoFisher scientific, Ulm, Germany) using the appropriate primers (**Table S1**). Melting temperatures of the primer were calculated by the online NEB Tm Calculator (http://tmcalculator.neb.com/#!/). After Identification of the PCR product on agarose gel, a SLG HighYield® Gel/PCR DNA extraction kit was used for DNA purification. The DNA concentrations were measured by a NanoDrop ND-2000c (ThermoFisher scientific, Ulm, Germany) spectrometer. For the in vitro BP recombinant reaction with BP Clonase™ II enzyme, 100 fmol of purified *att*B-PCR product and 50 fmol of *att*P-containing donor vector pDONR™ 201 in TE buffer were incubated over night at rt. The resulting *att*L-containing entry clone was transformed in chemically competent One Shot® TOP10 *E. coli* cells (Invitrogen) and the cells were plated on LB agar plates containing 25 µg mL^{-1} kanamycin. After selection of transformed cells, they were grown in LB medium, harvested and plasmids isolated using a HiYield® Plasmid Mini Kit (SLG). To generate the corresponding *att*B-containing expression clone, the LR Clonase™ II enzyme mix was added to 50 fmol of the *att*L-containing entry clone and 50 fmol of the *att*R-containing destination pDest007 vector. After transformation of the expression clone in chemically competent BL21 DE3 *E. coli* cells the clones were selected on LB agar plates containing 100 µg mL^{-1} carbenicillin. Validity of the clones was confirmed by plasmid sequence analysis.

For mutagenesis, the pDest plasmid containing the pqsD sequence was replicated with a forward primer that enclosed the desired mutation and a reverse primer with a phosphorylated 5´-end (**Table S1**) using the Phusion High-Fidelity PCR kit (ThermoFisher scientific, Ulm, Germany) according to the manufacturer's protocol. Afterwards the parental template plasmid was digested by DpnI endonuclease (NEB) and the PCR product was purified by agarose gel electrophoresis. The plasmid was ligated using T4 DNA ligase (NEB), transformed into chemically competent BL21 *E. coli* and clones were selected on LB agar plates containing 100 µg mL^{-1} carbenicillin. Restriction digest and ligation were conducted according to the manufacturer´s protocol. Validity of the clones was confirmed by plasmid sequence analysis.

For protein expression, 3 mL of an overnight culture of *E. coli* BL21 cells containing the recombinant clones were grown in LB medium (1000 mL) supplemented with carbenicillin (100 µg/mL) at 37 °C until an OD_{600} of 0.6. The temperature of the shaking incubator was reduced to 20 °C, then target gene expression was induced with anhydrotetracylin and after 18 h

incubation, bacterial cells were harvested by centrifugation and stored at −80 °C. On the next day the cells were thawed on ice, PBS was added, the suspension sonicated and the cell debris removed by centrifugation. The protein was purified by Strep-Tag affinity chromatography using a StrepTrap™ HP 5 mL column (GE Healthcare) and an ÄKTA™purifier system with UV detector (UPC 900, P900, Box900, Frac950, GE Healthcare). The purified protein was concentrated by an Amicon® Ultra-4 Centrifugal Filter Unit (Merck Millipore, Darmstadt, Germany) and stored in 10 µL aliquots at −80 °C. Concentrations of proteins were determined using a Bradford assay (Thermo Scientific).

Protein sequence of PqsD

MASWSHPQFEKGASTSLYKKAGYGNPILAGLGFSLPKRQVSNHDLVGRINTSDEFIVERTGVRTRYHVEP
EQAVSALMVPAARQAIEAAGLLPEDIDLLLVNTLSPDHHDPSQACLIQPLLGLRHIPVLDIRAQCSGLLYGL
QMARGQILAGLARHVLVVCGEVLSKRMDCSDRGRNLSILLGDGAGAVVVSAGESLEDGLLDLRLGADGN
YFDLLMTAAPGSASPTFLDENVLREGGGEFLMRGRPMFEHASQTLVRIAGEMLAAHELTLDDIDHVICHQ
PNLRILDAVQEQLGIPQHKFAVTVDRLGNMASASTPVTLAMFWPDIQPGQRVLVLTYGSGATWGAALYRK
PEEVNRPC

Protein sequence of PqsD C112A mutant (PqsDm)

MASWSHPQFEKGASTSLYKKAGYGNPILAGLGFSLPKRQVSNHDLVGRINTSDEFIVERTGVRTRYHVEP
EQAVSALMVPAARQAIEAAGLLPEDIDLLLVNTLSPDHHDPSQACLIQPLLGLRHIPVLDIRAQASGLLYGLQ
MARGQILAGLARHVLVVCGEVLSKRMDCSDRGRNLSILLGDGAGAVVVSAGESLEDGLLDLRLGADGNY
FDLLMTAAPGSASPTFLDENVLREGGGEFLMRGRPMFEHASQTLVRIAGEMLAAHELTLDDIDHVICHQP
NLRILDAVQEQLGIPQHKFAVTVDRLGNMASASTPVTLAMFWPDIQPGQRVLVLTYGSGATWGAALYRKP
EEVNRPC

(N-terminal strep-tag)

Table S1: Primer sequences of PqsD and PqsD C112A mutant (PqsDm)

	Primer sequence	T_m [°C]
PqsD	*forward* 5‘-GGGG ACA AGT TTG TAC AAA AAA GCA GGC TAC GGTAATCCGATCCTGGCCGG-3‘	63.5
	reverse 5‘-GGGG AC CAC TTT GTA CAA GAA AGC TGG GTG TCAACATGGCCGGTTCACCTCC-3‘	64
PqsDm	*forward* 5‘-CAGCGGGTTGCTGTACGGCTTG-3‘	74
	reverse 5‘-CCGGTACTGGATATCCGGGCACAG-3‘	74

2.3. Labeling experiments

All concentrations in labeling experiments were calculated for a total sample volume of 25 µL.

2.3.1. ABPP labeling and downscaling

ABP probe stock solutions in DMSO (0.6 µL) were added to mixtures containing PqsD or PqsDm (10 µg/mL) in reaction buffer (5 mM Tris, 10 mM KCl, pH 7.5). The solutions were incubated at 37 °C for 30 min. After incubation, click chemistry was performed according to the protocol.

2.3.2. NEM inhibition assay

To solutions of PqsD (10 µg/mL) in reaction buffer freshly prepared NEM stock solutions (0.6 µL) were added and incubated at rt for 2 h. Afterwards, probe **CA2** (0.6 µL, 200 µM in DMSO) was added and incubated at 37 °C for 30 min. After incubation, click chemistry was performed according to the protocol.

2.3.3. Competitive screening assay and dose down

To solutions of PqsD (10 µg/mL) in reaction buffer stock solutions of the compounds **1–9** or **10–16** in DMSO (0.6 µL) were added for described concentrations and pre-incubated at rt for 30 min. A vehicle control (reaction buffer with 0.6 µL DMSO) containing PqsD was treated equally.

Afterwards probe **CA2** (0.6 µL, 200 µM in DMSO) was added to each sample and incubated at 37 °C for 30 min. After incubation, click chemistry was performed.

2.3.4. Click control assay

For competitive samples, to solutions of PqsD (10 µg/mL) in reaction buffer stock solutions of the compounds **11**, **14**, **16** or **12** (0.6 µL, 10 mM in DMSO) were added and pre-incubated at rt for 30 min. A vehicle control (reaction buffer with 0.6 µL DMSO) containing PqsD was treated equally. Afterwards probe **CA1** (0.6 µL, 200 µM in DMSO) was added to each sample and incubated at 37 °C for 30 min. After incubation, click chemistry was performed. For click control samples, buffer stock solutions of the compounds **11**, **14**, **16** or **12** (0.6 µL, 10 mM in DMSO) were added directly before click reaction was started by adding 1 µL of $CuSO_4$ solution (25 mM in H_2O_{deion}).

2.3.5. Click chemistry

After incubation with the probes, 1 µL of Rhodamine azide stock (0.325 mM in DMSO), 1 µL of freshly prepared TCEP, tris(2-carboxyethyl)phosphine solution (26 mM, 15 g/L in H_2O_{deion}) and 1.5 µL of 1× TBTA Ligand stock (tris[(1-benzyl-1*H*-1,2,3-triazol-4-yl)methyl]amine) (1.67 mM in DMSO) were added. The solution was vortexed and the click reaction started by adding 1 µL of $CuSO_4$ solution (25 mM in H_2O_{deion}). The reaction was incubated at rt for 1 h. The reaction was stopped by addition of 25 µL of 2× SDS Gel loading buffer. The solution was stored at −18 °C until the analysis by SDS-PAGE.

2.3.6. Proteomics of PqsD

PqsD (1 mg/mL) in reaction buffer (50 µL) was incubated with 5 µL of **CA2** (2.5 mM in DMSO) for 30 min at 37 °C. Afterwards each sample was desalted using Nanosep 3K OMEGA centrifugal devices (Pall), digested with trypsin and measured on a Thermo LTQ Orbitrap Discovery with Eksigent 2D-nano HPLC (coverage 84.4%).

2.4. Coomassie gels

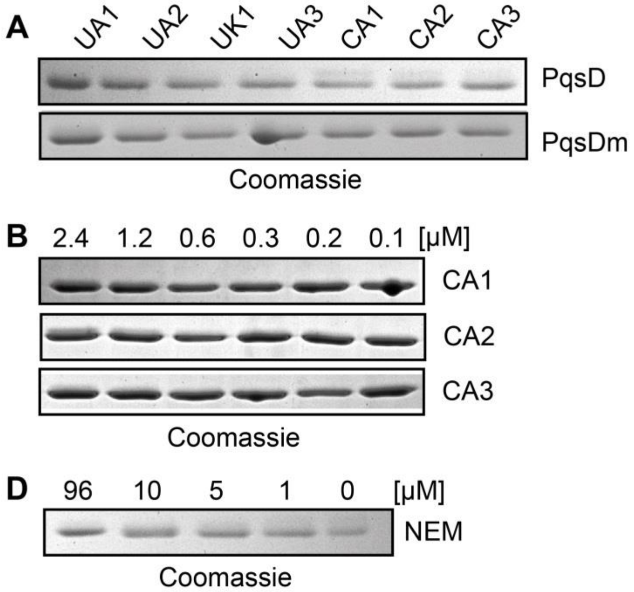

Figure S2: Corresponding coomassie staining of gels shown in Figure 3.

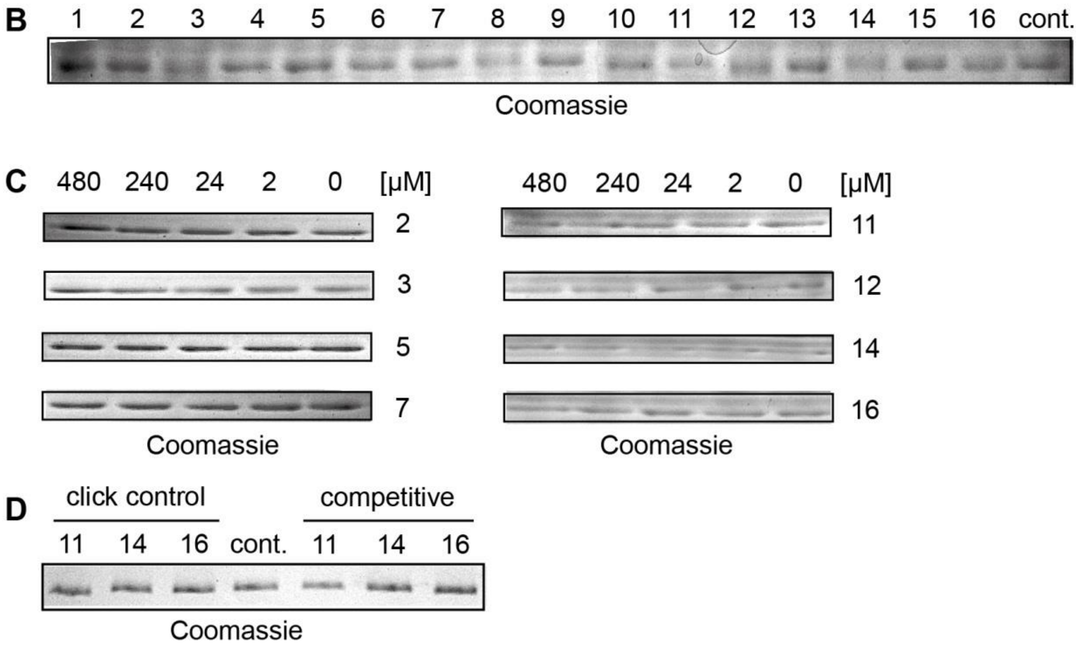

Figure S3: Corresponding coomassie staining of gels shown in Figure 5.

3. Studies to elucidate interaction of probe CA2 and inhibitors 11 and 14

NMR interaction study

The probe **CA2** and the compounds **11** or **14** were dissolved in THF (THF-d_6, 200 µL) and incubated for 1 h and 24 h at room temperature. After every time-point ^{1}H spectra of the mixture were recorded on a Bruker Avance III 400 instrument and calibrated on the residual solvent peak. Spectra were compared using MestReNova (Version: 10.0.2-15465, Mestrelab Research S.L.).

MS interaction study

The probe **CA2** and the compounds **11** or **14** were dissolved in THF/water (1:1) and incubated for 2 h or 18 h at rt. Afterwards the mixture was analyzed using an ESI-TOF (Bruker Daltonics microTOFII) equipped with a Chromolith FastGradient RP-18e 50×2 mm (Merck). LC-method with solvent A = water + 0.1% formic acid; B = acetonitrile + 0.1% formic acid: T_{0min} B = 5%; T_{2min} B = 5%; T_{7min} B = 100%; T_{12min} B = 100%; CA2: *m/z* = 146.0373 $[M+H]^+$ calc. for $C_6H_9ClNO^+$. Compound **11**: *m/z* = 276.1422 $[M+H]^+$ calc. for $C_{16}H_{22}NOS^+$. Compound **14**: *m/z* = 277.1263 $[M+H]^+$ calc. for $C_{16}H_{21}O_2S^+$.

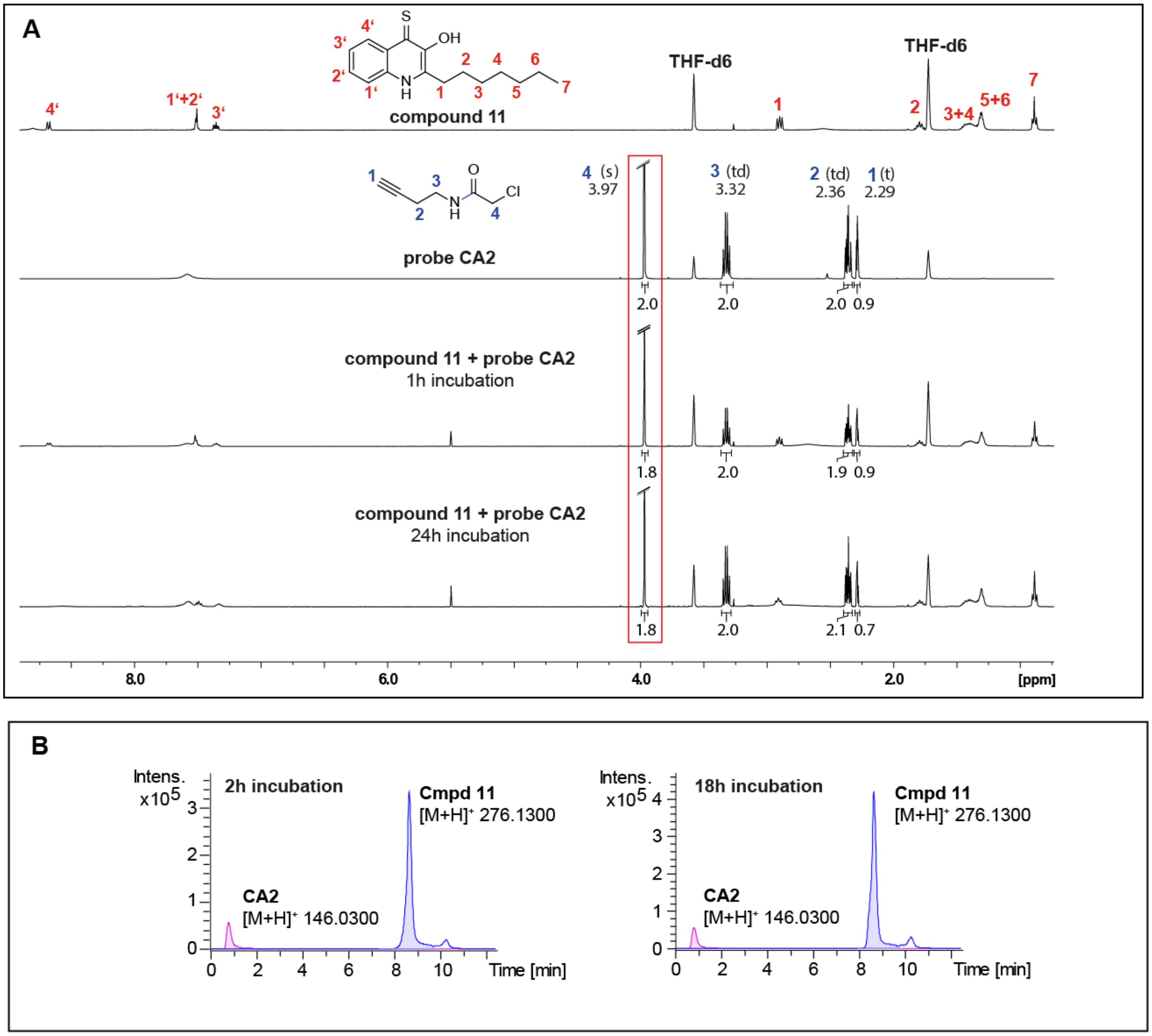

Figure S4: Stability of probe **CA2** with compound **11**. **A.** NMR analysis of a mixture containing compound **11** and probe **CA2** shows no interaction of both agents. **B.** ESI-MS analysis of the mixture show no significant change in the area of extracted ion chromatograms (EIC´s) after 18 h incubation.

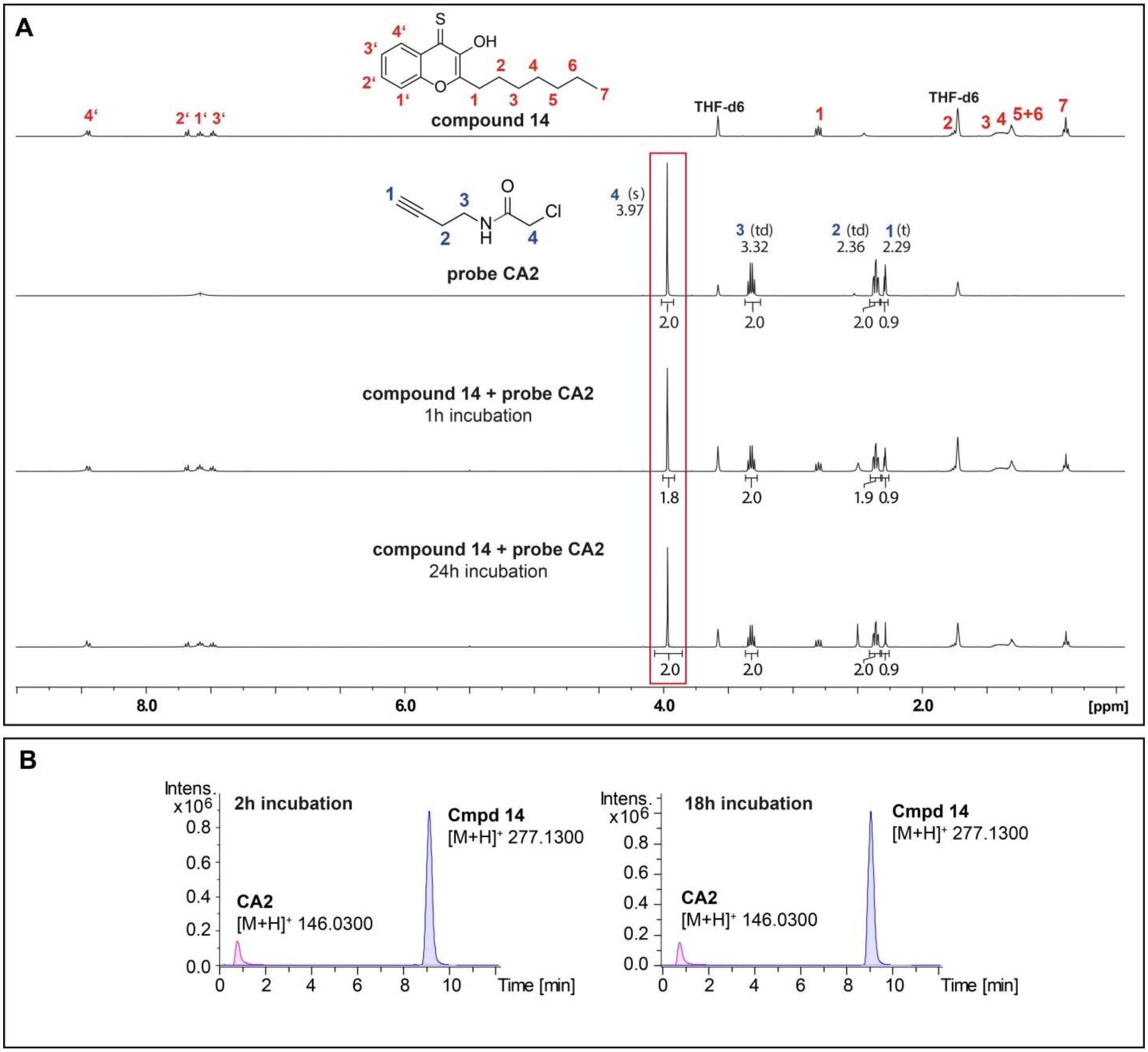

Figure S5: Stability of probe **CA2** with compound **14**. **A.** NMR analysis of a mixture containing compound **14** and probe **CA2** shows no interaction of both agents. **B.** ESI-MS analysis of the mixture show no significant change in the area of extracted ion chromatograms (EIC´s) after 18 h incubation.

4. References

1. Dadova, J., Orsag, P., Pohl, R., Brazdova, M., Fojta, M., Hocek, M., *Angewandte Chemie* **2013**, *52*, 10515-10518.
2. Weerapana, E., Simon, G. M., Cravatt, B. F., *Nature chemical biology* **2008**, *4*, 405-407.
